# Genomic Insights into Post‐Domestication Expansion and Selection of Body Size in Ponies

**DOI:** 10.1002/advs.202413023

**Published:** 2025-02-26

**Authors:** Xingzheng Li, Zihao Wang, Min Zhu, Binhu Wang, Shaohua Teng, Jing Yan, Haoyu Wang, Pengxiang Yuan, Shuwei Cao, Xiaolu Qu, Zhen Wang, Kai Zhan, Md. Panir Choudhury, Xintong Yang, Qi Bao, Sang He, Lei Liu, Pengju Zhao, Jicai Jiang, Hai Xiang, Lingzhao Fang, Zhonglin Tang, Yuying Liao, Guoqiang Yi

**Affiliations:** ^1^ Shenzhen Branch Guangdong Laboratory of Lingnan Modern Agriculture Key Laboratory of Livestock and Poultry Multi‐omics of MARA Agricultural Genomics Institute at Shenzhen Chinese Academy of Agricultural Sciences Shenzhen 518124 China; ^2^ Animal Husbandry Research Institute Guangxi Vocational University of Agriculture Nanning 530002 China; ^3^ Nanning Capitano Equestrian Club Co., Ltd Nanning 530000 China; ^4^ Anhui Provincial Key Laboratory of Livestock and Poultry Product Safety Institute of Animal Husbandry and Veterinary Medicine Anhui Academy of Agricultural Sciences Hefei 230031 China; ^5^ Hainan Institute Zhejiang University Yongyou Industry Park, Yazhou Bay Sci‐Tech City Sanya 572000 China; ^6^ Department of Animal Science North Carolina State University Raleigh NC 27695 USA; ^7^ Guangdong Provincial Key Laboratory of Animal Molecular Design and Precise Breeding School of Life Science and Engineering Foshan University Foshan 528225 China; ^8^ Center for Quantitative Genetics and Genomics Aarhus University Aarhus 8000 Denmark; ^9^ Kunpeng Institute of Modern Agriculture at Foshan Agricultural Genomics Institute at Shenzhen Chinese Academy of Agricultural Sciences Foshan 528226 China; ^10^ Guangxi Veterinary Research Institute Nanning 530001 China; ^11^ Bama Yao Autonomous County Rural Revitalization Research Institute Bama 547500 China

**Keywords:** body size, genome, pony, population genetics, regulatory elements

## Abstract

Horse domestication revolutionizes human civilization by transforming transportation, agriculture, and warfare patterns. Despite extensive studies on modern domestic horse origins, the intricate demographic history and genetic signatures underlying pony size remain unexplored. Here, a high‐quality genome assembly of the Chinese Debao pony is presented, and 452 qualified individuals from 64 horse breeds worldwide are extensively analyzed. The authors’ results reveal the conservation of ancient components in East Asian horses and close relationships between Asian horses and Western pony lineages. Genetic analyses suggest an Asian paternal origin for European pony breeds. These pony‐sized horses share close genetic affinities, potentially attributed to their early expansion and adaptation to local environments. In addition, promising *cis*‐regulatory elements influencing horse withers height by regulating genes such as *RFLNA* and *FOXO1* are identified. Overall, this study provides insightful perspectives on the dispersal history and genetic determinants underlying body size in ponies, offering broader implications for horse population management and improvement.

## Graphical abstract

The authors’ research delves into the history of ponies by assembling a pony genome and integrating horse genomes. This unveils genetic connections between Asian and European pony breeds. *Cis*‐regulatory elements of key genes impacting body size are also identified. Understanding these genetic nuances holds promise for enhancing the management of horse populations.

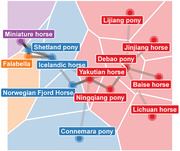

## 1 Introduction

The domestication of animals has been pivotal in shaping human civilization. Horses (*Equus caballus*) were first domesticated ≈5500 years ago on the Eurasian steppes.^[^ ^1^ ^]^ Their use in transportation, agricultural work, and warfare facilitated the expansion of human territories and the establishment of long‐distance trade routes.^[^ ^2^, ^3^, ^4^ ^]^ Among the diverse horse breeds, ponies are generally defined as horses standing less than 14.2 hands (58 in., 147 cm) at the withers. Bred for adaptability to various environments,^[^ ^5^ ^]^ ponies are found from the islands of Northern Europe to the mountainous regions of Southern China. Their hardiness, intelligence, and friendly nature have made them valuable for transportation, work, companionship,^[^ ^6^ ^]^ and recently, as popular mounts for children. Despite their significant roles, the genetic factors contributing to ponies’ unique characteristics remain understudied. Investigating the genetic architecture and diversity of pony breeds can offer insights into horse development history and trace patterns of human activity and civilization.

The Debao pony, a prominent local breed from Guangxi Province in Southern China, stands about 9.3 hands (39 in., 100 cm) at the withers and is believed to descend from ancient breeds existing 2000 years ago.^[^ ^7^ ^]^ While previous studies have explored the origins and spread of domestic horses from the Western Eurasian steppes,^[^ ^1^ ^]^ the post‐domestication development of horses, especially ponies, and their relationships remain unclear. The genealogy of pony clusters from Southern China among horse breeds is yet to be determined due to a lack of comprehensive datasets. Previous studies have identified candidate genes associated with body size in horses.^[^ ^8^, ^9^, ^10^, ^11^, ^12^ ^]^ However, these findings often focus on small subsets of breeds or regions. Integrative analyses that include Eurasian pony breeds could provide new avenues for understanding the genetic basis shaping pony characteristics and diversity.

To enhance our understanding of the complex demographic history and genetic signatures associated with pony body size, we assembled a high‐quality genome of the Debao pony using PacBio CLR, Illumina sequencing, and Hi‐C technologies. We then comprehensively analyzed whole‐genome data from 452 domesticated horses representing 64 breeds worldwide to gain insights into the development and breeding history of ponies. By integrating genetic data with 3D genome and histone mark ChIP‐seq datasets, we uncovered novel genetic factors influencing horse withers height. This study provides new perspectives on the post‐domestication history of horse development and has important implications for the management and improvement of modern horse populations.

## 2 Results

### 2.1 Genome Assembly and Annotation of the Debao Pony Genome

**Figure 1:**
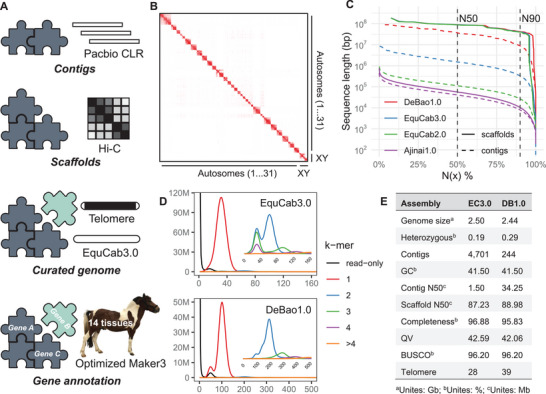
Genome assembly and annotation of the Debao pony. A) Schematic diagram illustrating the pipeline used for genome assembly and annotation. B) Hi‐C chromatin interaction map of the assembled Debao pony genome. C) Comparison of assembly contiguity between currently available horse genomes and the DeBao1.0 assembly. D) Copy number spectra (spectra‐cn) of k‐mers for EquCab3.0 and DeBao1.0. Enlarged views of k‐mers with copy numbers greater than 1 (2, 3, 4, >4) are provided within each plot. E) Assembly statistics of EquCab3.0 (EC3.0) and DeBao1.0 (DB1.0). The contig N50 was calculated by breaking the genome into contigs at gap regions.

We assembled a high‐quality, chromosome‐level genome of the Debao pony by integrating PacBio continuous long reads (CLR) (296 Gb, 121×), Illumina paired‐end (PE) reads (333 Gb, 136×), and Hi‐C sequencing data (119 Gb) (**Figure** **1**A; Figure S1, Supporting Information). The estimated genome size was 2.44 Gb with a heterozygosity rate of 0.29% (Figure S2 and Table S1, Supporting Information). Using Canu to assemble the PacBio long reads (Figure S3, Supporting Information), we initially obtained 583 contigs with an N50 of 20.48 Mb after purging haplotigs (Table S2, Supporting Information). The resulting chromosome‐level assembly then underwent manual curation and polishing to enhance accuracy (Figure S4, Supporting Information), with rigorous quality checks at each step (Figure S5, Supporting Information). The Hi‐C chromatin interaction heatmap demonstrated clear genomic interactions, indicating high assembly quality and accuracy (Figure 1B; Figure S6, Supporting Information). The final genome assembly spanned 2.44 Gb, comprising 31 autosomes, X and Y sex chromosomes, a mitochondrial genome, and 210 unassigned contigs. It achieved a contig N50 of 34.25 Mb and a scaffold N50 of 88.98 Mb (Figure 1C; Table S2, Supporting Information). Notably, our Debao pony assembly rectified uncollapsed artificial duplications present in EquCab3.0 (Figure 1D), exhibiting high contiguity and completeness compared to other released *E*. *caballus* genomes (Figure 1C,E; Figure S7 and Table S2, Supporting Information).

**Figure 2:**
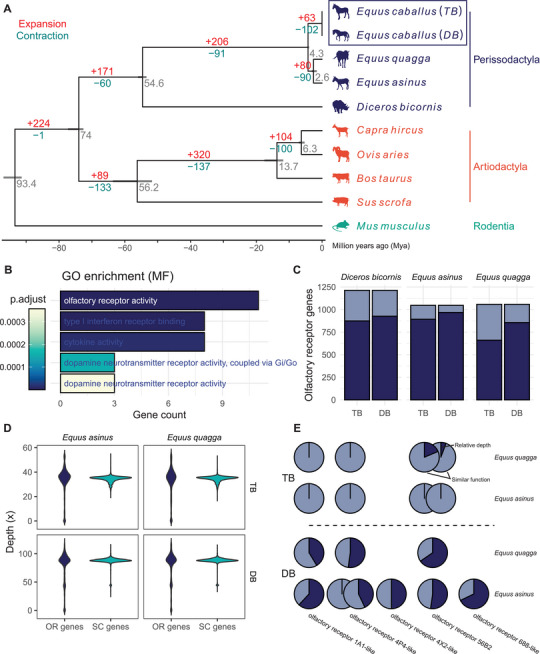
Evolution of the olfactory receptor gene family in *E. caballus*. TB represents the EquCab3.0 assembly of the Thoroughbred and DB represents the DeBao1.0 assembly of the Debao pony. A) Phylogenetic tree and gene family evolution of *E. caballus* among nine species. The numbers of gene gains and losses are indicated on the corresponding branches. Divergence time intervals are represented by light grey bars at the internodes, with estimated divergence times provided beside each bar. B) The top five enriched gene ontology (GO) molecular function (MF) terms for the contracted gene family in *E. caballus* (indicated by the rectangle in panel A). C) Detected olfactory receptor genes of other *Perissodactyla* species (*D. bicornis, E. asinus, and E. quagga*) in *E. caballus* (TB and DB). Olfactory receptor genes detected in *E. caballus* are represented in dark blue. D) Violin plot of sequencing depth for olfactory receptor (OR) genes and single‐copy (SC) genes annotated in *E. asinus* and *E. quagga*, using aligned reads from *E. caballus* (TB and DB). E) Putative lost olfactory receptor genes in *Equus caballus*. Each circle represents an olfactory receptor gene, with genes in the same column having similar functions. The dark blue sector in each circle represents the ratio of the sequencing depth of the olfactory receptor gene to the average depth of single‐copy genes (relative depth). These putative lost olfactory receptor genes were detected by aligning *E. caballus* (TB and DB) sequencing data to the genomes of *E. asinus* and *E. quagga*, respectively. They were validated within the constrained olfactory receptor gene family identified through comparative genomic analysis in horses.

Repetitive sequences accounted for 41.26% of the Debao pony genome (Table S3, Supporting Information). Using an optimized MAKER annotation pipeline, we predicted a total of 21 038 high‐confidence protein‐coding genes (Figures S8–S10, Supporting Information). These gene models were further refined using Apollo for enhanced accuracy. Interestingly, utilizing the assemblies of the Debao pony and Thoroughbred horse genomes as representatives of *E. caballus*, we discovered a significant loss of genes associated with olfactory receptor activity, a phenomenon observed across diverse horse breeds (**Figure** **2**A,B).^[^ ^13^ ^]^ The contraction of the olfactory receptor gene family, observed through comparative genomic analysis, was further validated by mapping sequences from other *Perissodactyla* species to horse genomes and by aligning horse sequencing reads to the olfactory receptor genes of related species (Figure 2C–E). These analyses confirmed that the reduced number of olfactory receptor genes in horses is not an artifact of genome assembly or annotation but rather reflects a genuine biological phenomenon. This suggests that the olfactory receptor gene family may have undergone relaxed selective constraints during horse domestication or as a result of divergence between horses and other equids. Overall, the high quality of the Debao pony genome establishes it as a valuable resource for genetic research in horses.

### 2.2 Genome‐Wide Variation and Population Structure of *E*. *caballus*

**Figure 3:**
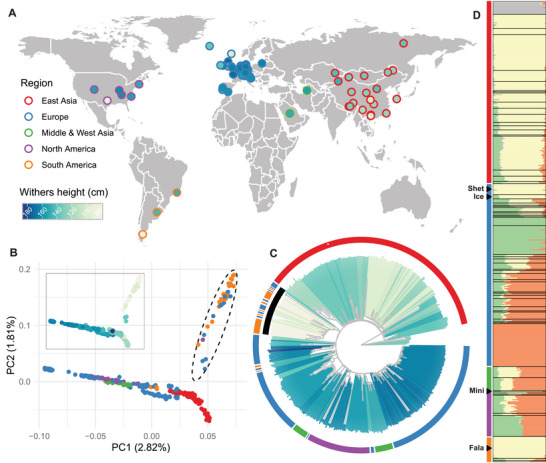
Geographic distribution and population structure of *E. caballus*. A) Geographic origin of 64 horse breeds included in the study. Colors representing regions and withers height are consistent throughout the study. B) Principal component analysis (PCA) plot showing the genetic relationships among horse populations. The black dashed ellipse highlights the Western pony lineage, which includes the Shetland pony, Icelandic horse, Miniature horse, and Falabella. Colors in the main plot and the thumbnail (top left) indicate regions and withers height, respectively. C) Neighbor‐joining (NJ) tree, rooted on Przewalski's horses, depicts the genetic clustering of horse populations. The black inner ring highlights the Western pony lineage, including the Shetland pony, Icelandic horse, Miniature horse, and Falabella. Colors of the outer ring indicate regions; while, branch colors correspond to withers height. D) Admixture analysis (*K* = 4) of horse populations, revealing genetic contributions from different ancestral sources. Breeds are ordered by regions, with colored strips on the left indicating these regions (e.g., East Asia, Europe). The bars on the right display the genetic composition of each population, with different colors representing distinct ancestral components. “Shet,” “Ice,” “Mini,” and “Fala” are abbreviations for Shetland pony, Icelandic horse, Miniature horse, and Falabella, respectively.

To comprehensively understand the genetic architecture of ponies across horse populations, we re‐sequenced ten Debao ponies, ten Baise horses, and six Warmblood horses at an average depth of ≈10.87×. In addition, we collected 476 previously sequenced horse genomes to capture greater genetic diversity. Using the genome analysis toolkit (GATK) and our high‐quality assembly as a reference, we identified a total of 6 14 72 656 single nucleotide polymorphisms (SNPs) throughout the genome (Figure S11 and Tables S4 and S5, Supporting Information). Of these SNPs, 59.20% were located within intergenic regions and 28.47% within introns (Table S6, Supporting Information). After excluding potential crossbred samples, we compiled a dataset of 452 high‐quality samples representing 64 horse breeds worldwide (**Figure** **3**A; Tables S7 and S8, Supporting Information).

We utilized all eligible SNPs to investigate the population structure and genetic diversity within the horse population. Principal component analysis (PCA) revealed geographic clustering and a spectrum reflecting the variation in withers height (Figure 3B; Table S9, Supporting Information). Notably, horses in East Asia were generally pony‐sized (withers height <147 cm), and ponies from other regions, especially those belonging to the lineage including the Shetland pony, Icelandic horse, Miniature horse, and Falabella, exhibited closer relationships with East Asian horses. The neighbor‐joining (NJ) phylogenetic tree displayed similar classification patterns, albeit with some scattered geographic clustering due to complex breeding histories (Figure 3C). The pony lineage comprising the Shetland pony, Icelandic horse, Miniature horse, and Falabella was phylogenetically closer to East Asian horses. These findings were strongly supported by admixture analysis results. At a *K* value of 4, the Western pony lineage predominantly shared more ancestry components with Asian horses than with European horses (Figure 3D; Figure S12, Supporting Information). With higher *K* values, these pony lineages separated from Asian horses and formed a distinct group (Figure S13 and Table S10, Supporting Information). Our analysis reveals the genome‐wide variation and population structure of *E. caballus*, providing novel insights into the close genetic relationship between this specific European pony lineage and East Asian horses.

### 2.3 Y Chromosome Haplotypes (HTs) and Phylogenetic Analysis

**Figure 4:**
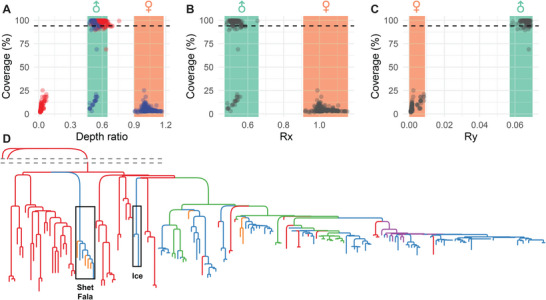
Characterization of the Y chromosome in stallions. A) Sequencing depth versus read coverage on the Y chromosome for each individual. Blue and red points represent the sequencing depth ratio of ChrX/Chr1 and ChrY/Chr1 for each individual, respectively. The black dashed line indicates the threshold of read coverage used to determine male individuals. The two rectangular sections correspond to male and female individuals based on the sequencing depth ratio of ChrX to Chr1. B) Rx index versus read coverage on the Y chromosome. C) Ry index versus read coverage on the Y chromosome. D) Clustering based on variations observed in the Y chromosome. “Shet,” “Fala,” and “Ice” refer to Shetland pony, Falabella, and Icelandic horse, respectively.

To explore the paternal genetic history of pony populations, we analyzed Y chromosome haplotypes and performed a phylogenetic study on 201 male individuals from our horse dataset (**Figure** **4**A; Table S11, Supporting Information). Male individuals were identified by detecting sufficient read coverage on the Y chromosome, enhancing the effective use of SNP data. We further validated these identified males using the Rx and Ry indices (Figure 4B,C), which allowed us to correct misassigned sex information for ten samples (Table S12, Supporting Information). In total, we identified 148 Y chromosome haplotypes across these horses (Table S13, Supporting Information). Notably, we found that the haplotypes of the Icelandic horse, Shetland pony, and Falabella resided within the East Asian clade (Figure S14, Supporting Information). Phylogenetic analysis indicated that the paternal lineages of the Shetland pony and Icelandic horse were primarily descended from horses of Asian origin, suggesting an Eastern paternal heritage (Figure 4D). Overall, our analysis of paternal lineage ancestry contributes to a deeper understanding of the genetic diversity and evolutionary history of ponies.

### 2.4 Phylogenetic Reconstruction and Genetic Affinities of Ponies

**Figure 5:**
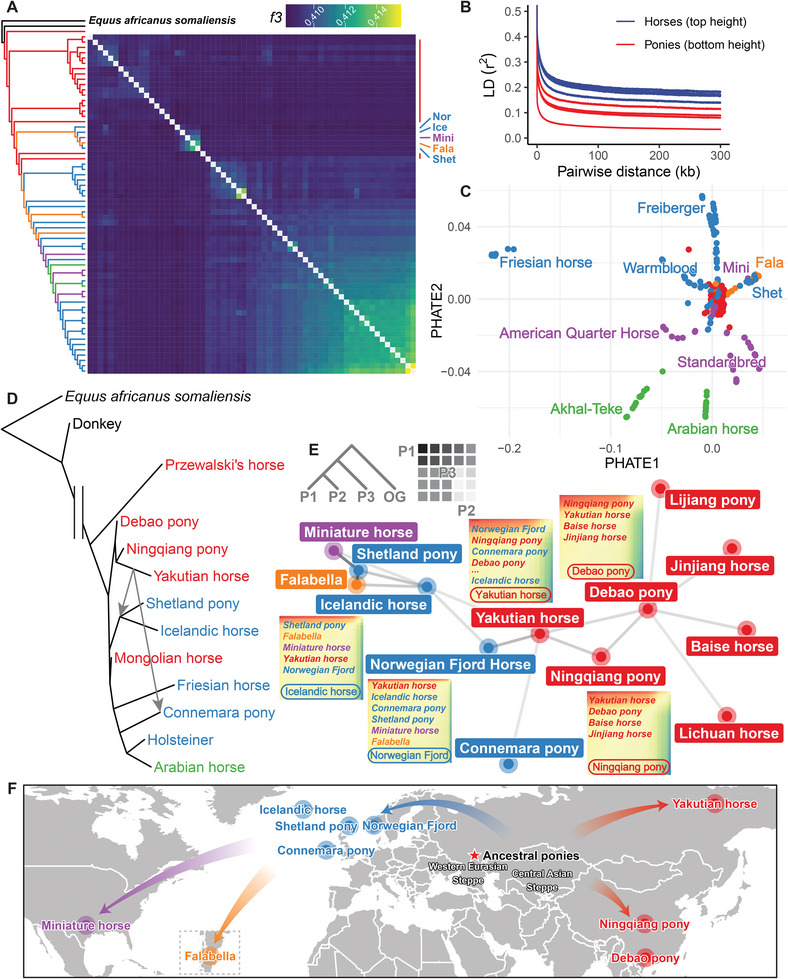
Genetic affinities of ponies in the horse population. A) TreeMix phylogenetic relationships and outgroup *f3*‐statistics (*E. caballus*, *E. caballus*; *E. africanus somaliensis*) of the horse population. Abbreviations denote the positions of the Norwegian Fjord Horse (“Nor”), Icelandic horse (“Ice”), Miniature horse (“Mini”), Falabella (“Fala”), and Shetland pony (“Shet”). B) Comparison of linkage disequilibrium (LD) decay patterns between the tallest horse breeds (Oldenburger, Holsteiner, Hanoverian horse, and Warmblood) and the smallest pony breeds (Baise horse, Shetland pony, Debao pony, and Falabella). C) PHATE plot depicting the distinct clustering of various horse breeds. D) Phylogeny and inferred mixture events of representative breeds as determined by TreeMix. E) Pony‐related genetic network based on all possible combinations of *f4*‐statistics (P1, P2; P3, Outgroup). Line colors, ranging from light to dark, indicate the strength of genetic relationships as revealed by *f4*‐statistics, from distant to close. The top left inset shows the basic *f4*‐statistics configuration. Surrounding the main network are example *f4*‐statistics heatmaps for P3, featuring the Yakutian horse, Norwegian Fjord Horse, Icelandic horse, Debao pony, and Ningqiang pony. The most genetically related breeds for each P3 are listed starting from the top left of each heatmap. F) Modeled dispersal of ponies inferred from sequencing data and historical knowledge. The red star marks the inferred center of horse domestication.

To further assess the extent of shared genetic drift among different horse populations, we performed phylogenetic reconstruction and outgroup *f3*‐statistics analysis. Recognizing the Norwegian Fjord Horse as one of the oldest and purest breeds in Northern Europe, with characteristics that bridge Western pony breeds, we included a Norwegian Fjord Horse sample in this analysis and subsequent *f4*‐statistics calculations. Heatmaps generated from the outgroup *f3*‐statistics indicated an early divergence of Asian horses, followed by Western ponies, aligning with our population structure findings (**Figure** **5**A; Figures S15 and S16, Supporting Information). The rapid linkage disequilibrium (LD) decay observed in ponies also suggests a potentially more primitive state (Figure 5B). To capture the global relationships and developmental trajectories of various breeds, we calculated the likelihood of transition between states by employing the potential of heat diffusion for affinity‐based transition embedding (PHATE). The PHATE‐embedded data generated nine major linear trajectories representing divergent genetic states, extending outward from the central region (Figure 5C; Table S14, Supporting Information). Notably, Western ponies formed one of these primary trajectories. In addition, East Asian horses, rather than European ones, were located in the adjacent basal region, reflecting transitional genetic states between Western ponies and their wild ancestors. To investigate potential gene flow among pony lineages, we selectively analyzed representative breeds with long breeding histories using TreeMix. The analysis inferred gene flow from the Yakutian horse to the predecessors of the Shetland pony and Icelandic horse, as well as to the Connemara pony (Figure 5D; Table S15, Supporting Information). To further elucidate gene flow patterns underlying pony domestication and breeding history, we calculated *f4*‐statistics for every possible combination across all sequenced breeds and constructed a pony relationship network based on key *f4*‐statistics values (Figure S17, Supporting Information). This network revealed that gene flows and connections between Western and Eastern ponies were essentially mediated by the Yakutian horse (Figure 5E; Figure S18, Supporting Information). Considering geographic distances and existing knowledge about the Yakutian horse, we propose that most modern ponies descended from an ancient pony‐sized lineage closely related to the Yakutian horse. Altogether, we hypothesize that the spread of contemporary ponies primarily originated from a shared ancestral pony population within the DOM2 lineage, likely residing in the Eurasian steppe (Figure 5F).

### 2.5 Novel Selection Signatures Associated with Horse Withers Height

**Figure 6:**
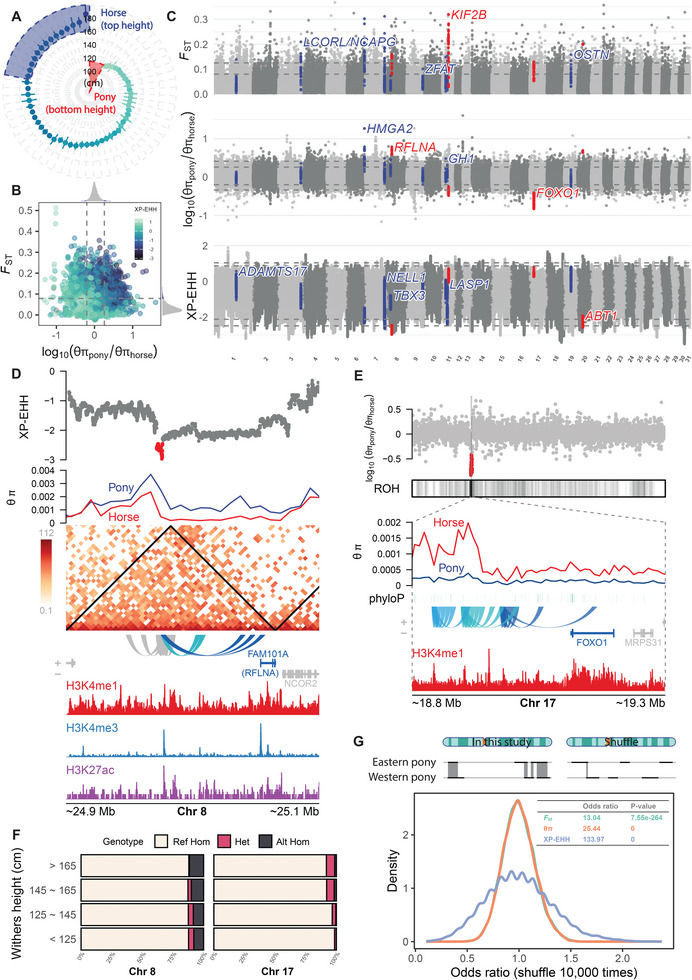
Selective regions associated with horse withers height. A) Breeds representing large‐sized horses and small‐sized ponies. Large horse breeds (top height): Shire horse, Clydesdale horse, Percheron, Oldenburger, Holsteiner, Württemberger, Irish Sport Horse, Hanoverian horse, Dutch Warmblood, Belgian Warmblood, Westphalian horse, Bavarian Warmblood, Warmblood, and Trakehner. Small pony breeds (bottom height): Ningqiang pony, Shetland pony, Debao pony, Miniature horse, and Falabella. Points denote the average withers height for each breed, with whiskers representing the range. B) Overview of selective signals related to horse body size, incorporating *F*_ST_, π ratio, and XP‐EHH (averaged over 10 kb windows). Grey dashed lines indicate the top 5% quantiles for *F*_ST_ and π ratio; while, XP‐EHH values are color‐coded. C) Detailed genome‐wide selective signals for horse body size based on *F*_ST_, π ratio, and XP‐EHH (pony vs horse). Grey dashed lines highlight the top 1% and 5% quantiles. Red points represent candidate regions, and blue points mark previously reported gene regions associated with horse body size. D) Candidate selective region on chromosome 8. Tracks, from top to bottom, display XP‐EHH (pony vs horse), *θ*π, Hi‐C contact matrix, genomic interactions, gene models, and histone ChIP‐seq signals (diaphysis of the metacarpal bone). Topologically associating domains (TADs) are marked by black triangles in the Hi‐C matrix. E) Candidate selective region on chromosome 17. Tracks, from top to bottom, display π ratio (*θ*π_pony_/*θ*π_horse_), ROH_pony_, *θ*π, phyloP conservation scores, genomic interactions, gene models, and histone ChIP‐seq signals (diaphysis of metacarpal bone). F) Genotype frequencies of candidate regions on chromosomes 8 and 17 across different withers heights. G) Fisher's exact test results showing the overlap of selective regions between Eastern pony versus horse and Western pony versus horse, based on *F*_ST_, π ratio, and XP‐EHH (pony vs horse). The odds ratio and P‐value are displayed in the top right. The main plot shows a density plot of the odds ratio, shuffled 10 000 times. Above the main plot, a schematic illustrates the overlaps of selective regions before and after the shuffling, with black lines indicating selective regions and grey rectangles representing overlapped regions. *P*‐values too small to be computed by the software are reported as zero.

To decipher the genetic basis of body size variation in horses, we performed a comprehensive genome‐wide selection analysis. We focused on pony breeds (withers height <1.02 m) from both Western and Eastern lineages and 14 horse breeds (withers height >1.65 m) with divergent withers heights (**Figure** **6**A). By integrating multiple selection metrics including fixation index (*F*_ST_), nucleotide diversity (π) ratio, and cross‐population extended haplotype homozygosity (XP‐EHH), we identified numerous candidate genomic regions and associated genes (Figure 6B; Tables S16–S18, Supporting Information). Among these candidate regions, we confirmed the involvement of genes previously linked to horse body size, such as high‐mobility group *AT‐hook 2* (*HMGA2*) and *NEL‐like 1* (*NELL1*). In addition, our analysis revealed four novel candidate genes: refilin A (*RFLNA*), kinesin family member *2B* (*KIF2B*), forkhead box O1 (*FOXO1*), and activator of basal transcription 1 (*ABT1*), which may play roles in regulating horse withers height (Figure 6C).

One notable selective region encompasses a regulatory element upstream of the *RFLNA* gene, known to influence bone mineralization, bone maturation, and chondrocyte development. This region spans ≈5.89 kb on chromosome 8 (Figure 6D). It exhibited the lowest XP‐EHH values (pony vs horse) across chromosome 8, suggesting positive selection toward larger body size in horses. Moreover, the reduction in nucleotide diversity within horse populations in this genomic region further supports its role in body size selection. We integrated ChIP‐seq data for three histone modifications (H3K4me1, H3K4me3, and H3K27ac) from the Functional Annotation of Animal Genomes (FAANG) database, focusing on bone tissue due to its relevance to skeletal development. The regulatory region near *RFLNA* exhibits enrichment for H3K4me1 and H3K27ac, characteristic of active enhancers, as well as H3K4me3. Hi‐C chromatin interaction data revealed that this region resides within the same topologically associating domain (TAD) as *RFLNA*, suggesting close spatial proximity and potential regulatory interactions. This combination of histone marks and chromatin architecture supports the role of this region as an enhancer for *RFLNA*, potentially influencing gene expression related to body size in horses.

Another identified selective region harbored multiple enhancers located downstream of the *FOXO1* gene, a known regulator of osteoblast numbers, bone mass, and chondrogenic commitment of skeletal progenitor cells, spanning 18.79 to 18.94 Mb on chromosome 17 (Figure 6E). The presence of reduced nucleotide diversity and extended runs of homozygosity (ROH) in the pony population suggests that this region has been conserved across most pony breeds, emphasizing its significance in determining small body size (Figure S19, Supporting Information). These enhancers, positioned close to *FOXO1*, may synergistically regulate its expression. High phyloP scores surrounding these enhancers further support their critical role in *FOXO1* regulation.

In addition to these examples, several other selective signals were associated with body size variation (Figures S20 and S21, Supporting Information). Given that body size is a polygenic trait, we demonstrated differences in allele frequency spectra at candidate loci across four distinct body size groups. The gradient in allele frequencies across these selective regions, in concordance with withers height, strongly indicates their involvement in regulating horse body size (Figure 6F; Figure S22, Supporting Information). These findings underscore the significance of regulatory elements adjacent to candidate genes in contributing to the observed variation in horse body size.

Further, we evaluated the overlap of selective regions between Eastern pony versus horse and Western pony versus horse. Our analysis revealed substantial overlap in the selective regions identified in both comparisons. The calculated odds ratios for *F*_ST_, π ratio, and XP‐EHH were magnitudes higher (by ten‐ to thousand‐fold) than those from randomly shuffled permutations, providing strong statistical support for the observed overlap (Figure 6G). These high odds ratios further underscore the close genetic relationship between Eastern and Western ponies.

## 3 Discussion

The origin and domestication of modern horses have long intrigued researchers.^[^ ^14^, ^15^, ^16^ ^]^ The Western Eurasian steppes, particularly the lower Volga‐Don region, are identified as the probable domestication center of modern domestic horses, known as DOM2, dating back to around 3000 BC.^[^ ^1^ ^]^ By ≈2200 BC, these horses began their global expansion,^[^ ^17^ ^]^ leading to the emergence of diverse horse breeds with a wide range of body sizes, from less than 1 meter to nearly 1.8 meters at the withers. Among these horse breeds, ponies have gained worldwide popularity due to their small size, versatility, appeal across age groups, accessibility, and therapeutic benefits.^[^ ^6^ ^]^ However, their genetic relationships and breeding history have remained largely unexplored.

To gain valuable insights into the development history and specific characteristics of ponies, we assembled a high‐quality genome of the Debao pony and conducted comprehensive analyses using global sequencing data. Unlike EquCab3.0, which utilized short‐read data,^[^ ^18^ ^]^ we employed PacBio long‐read sequencing to construct the genome backbone of the Debao pony assembly. This approach significantly improved contiguity, achieving a 20‐fold increase in the contig N50 value and resulting in fewer gaps across the genome. We also achieved base accuracy comparable to that of EquCab3.0, which was based on Sanger sequence data.^[^ ^18^ ^]^ Importantly, our assembly also addressed issues of artificial duplications overrepresented in EquCab3.0, which can complicate read alignment and variant calling, potentially causing overestimated gene family expansions.^[^ ^19^ ^]^ The resulting high‐quality pony genome enhances our understanding of horse genetic diversity and provides the scientific community with a valuable resource for equine genetics and genomics. Moving forward, the Debao pony genome, along with other horse assemblies, can be utilized in large‐scale pan‐genome analyses. Such studies would not only enrich the genome‐wide genetic variation but also shed light on the biological adaptability across diverse horse breeds.^[^ ^20^ ^]^

Our population structure analysis revealed that the Western pony lineage, comprising the Shetland pony, Icelandic horse, Miniature horse, and Falabella, exhibited closer genetic relationships to Eastern horses. This suggests potential historical connections and gene flow between these pony breeds and Eastern horse populations. Historically, horses have been transported and traded through various means, including invasion, migration, and commerce.^[^ ^21^, ^22^, ^23^ ^]^ Evidence indicates that early breeders likely selected stallions based on visible phenotypic traits for breeding purposes.^[^ ^24^ ^]^ Delving deeper into the genetic history, we investigated Y chromosome lineages, providing compelling evidence supporting an Eastern paternal origin for this European pony lineage. East Asia, particularly the southwestern region of China, has preserved a rich diversity of Y chromosomal haplotypes and ancient paternal horse lineages.^[^ ^25^ ^]^ Recent studies have indicated their foundational position within the non‐Crown haplogroups.^[^ ^26^ ^]^ In contrast, Europe has experienced a decline in genetic diversity among ancient domestic stallions over the past 4000 years. Notably, the Y‐HT‐1 haplotype, exhibited by present‐day horses, has replaced most European haplotypes, except in Yakutian horses, suggesting a higher diversity of patrilineages originating from further East.^[^ ^27^, ^28^, ^29^ ^]^ However, limited ancient materials have been preserved or discovered to reveal the landscape of ancient horse Y chromosomes in East Asia. In our research, we further elucidated ancient components of East Asian horses and demonstrated a genetic connection between European and Asian ponies through Yakutian horses. Contemporary Yakutian horses were most likely introduced following the migration of the Yakut people from the Altai‐Sayan or Baïkal area between the 13th and 15th centuries or even earlier.^[^ ^30^ ^]^ Despite the absence of historical records detailing the Yakutian horse's predecessors, their rapid adaptation and geographical isolation significantly contributed to the preservation of ancestral genetic components within their genome.

Reconstructing the historical trajectory of ponies from their early formation period presents significant challenges. Warhorses, known for their large size and high speed, facilitated the spread of humans and horse breeds through warfare.^[^ ^31^ ^]^ In contrast, pony‐sized horses lack the same physical attributes or speed advantages. Therefore, the breeding history and dispersal routes of ponies may be more complex and obscured by the intricate relationships among horse populations. Previous studies estimate that the Shetland pony and Icelandic horse originated over 1000 years ago, accompanying the arrival of Norse settlers during the Viking Age.^[^ ^32^ ^]^ Eastern pony breeds such as the Debao pony are believed to descend from the ancient Guoxia breed, which dates back thousands of years. However, the exact origins of their ancestors remain elusive. Based on our comprehensive analyses, we speculate that they originated from an ancestral pony population within the DOM2 lineage, likely centered around the Eurasian steppe. The absence of direct ancient DNA samples from this ancestral population, along with its close affinity to extant Yakutian horses, results in the current genetic relationship network observed in our study. Interestingly, these pony‐sized breeds are often located on islands or in mountainous regions, where geographical isolation has limited gene flow, leading to their basal placement in phylogenetic trees. For instance, the Shetland pony and Icelandic horse, both island‐based breeds with relatively small populations, may experience pronounced genetic drift and founder effects. This phenomenon can exaggerate their basal position in phylogenetic analyses. Expanding the sample size of such isolated breeds in future studies can mitigate this effect by capturing additional genetic diversity, thereby refining phylogenetic inferences.^[^ ^33^ ^]^ Given that previous studies have predominantly focused on European horses,^[^ ^27^ ^]^ our findings extend the understanding of ancient genetic components and diversity in East Asian horses. To fully comprehend the breeding history of ponies and other horse breeds post‐domestication, additional ancient genomes covering a broader geographical spectrum, especially from East Asia and local rural regions, will be crucial. Such data would help clarify the complex demographic events and migrations that shaped the genetic landscape of modern ponies.

While previous work has identified candidate genes associated with body size, most studies have focused on specific breeds or regions and often relied on low‐density SNP chip datasets.^[^ ^9^, ^11^ ^]^ By integrating a large‐scale resequencing dataset spanning horses of widely varying withers heights, we uncovered previously unknown genetic information related to body size. For instance, frameshift variants in *RFLNA* have been linked to spondylocarpotarsal synostosis syndrome, a skeletal disorder characterized by short stature and carpal/tarsal synostosis.^[^ ^34^ ^]^ The involvement of *FOXO1* in embryonic development, bone growth and remodeling, and cartilage repair processes underscores its essential function in skeletal development.^[^ ^35^ ^]^ Another signal was identified near the *KIF2B* gene, which belongs to the KIF family involved in nervous system development and early embryo. In addition, a selective signal encompassed regulatory elements of the *ABT1* gene, which plays a critical role in growth and height traits.^[^ ^36^ ^]^ An interesting finding is that most candidate regions were located within *cis*‐regulatory elements, underscoring the significant role of transcriptional evolution in driving rapid adaptation and the breed formation process. These newly discovered *cis*‐regulatory elements not only provide insights into the function of noncoding regions but also deepen our understanding of the regulation of horse height. Further functional characterization and validation of these genes and regulatory elements will enhance our understanding of the molecular mechanisms driving withers height variation in horses.

## 4 Conclusion

In conclusion, we assembled and annotated a high‐quality genome of the Debao pony, providing a valuable resource for equine research. Through comprehensive genetic analyses, we gained profound insights into the intricate genetic relationships among various pony breeds. In addition, our investigation into horse withers height led to the discovery of novel *cis*‐regulatory elements involved in this trait. These findings enhance our understanding of pony genetics and offer a foundation for future studies on breed characteristics and trait selection.

## 5 Experimental Section

### Ethics Statement

This study adhered to the ethical guidelines outlined in the “Guide for the Care and Use of Experimental Animals” established by the Ministry of Agriculture and Rural Affairs (Beijing, China). The protocols were reviewed and approved by the Institutional Animal Care and Use Committee of both the Chinese Academy of Agricultural Sciences and the Guangxi Veterinary Research Institute. To minimize any potential suffering, the horses were humanely euthanized prior to tissue sampling when required.

### Genome Assembly

A male Debao pony from Debao County, Baise, Guangxi Province, China, was selected for genome assembly. Genomic DNA was extracted from its blood using the DNeasy Blood & Tissue Kit (Qiagen, Beijing, China), and DNA quality was assessed via agarose gel electrophoresis. For long‐read sequencing, single‐molecule real‐time (SMRT) PacBio sequencing libraries were prepared following standard Pacific Biosciences protocols and sequenced on the PacBio Sequel platform. Hi‐C libraries were constructed using the Phase Genomics Proximo Hi‐C Animal Kit (Phase Genomics, Washington, USA). According to the manufacturer's instructions, chromatin was cross‐linked with formaldehyde, digested with the DpnII restriction enzyme, end‐repaired, and proximity‐ligated to form chimeric junctions. The Hi‐C libraries were sequenced on an Illumina NovaSeq 6000 platform. In addition, short‐read paired‐end (PE) sequencing libraries were prepared and sequenced on the Illumina NovaSeq 6000 platform.

The genome assembly followed the vertebrate genomes project (VGP) assembly pipeline,^[^ ^37^ ^]^ with specific modifications (Figure S1, Supporting Information). The genome size was estimated by analyzing the frequency distributions of k‐mers (sizes 17, 19, 21, 23, 25, 27, 29, and 31) from Illumina PE reads using Jellyfish (v2.2.10)^[^ ^38^ ^]^ and calculated using GenomeScope (v2.0).^[^ ^39^ ^]^ The PacBio reads were independently assembled into contigs using three different assemblers: FALCON (v0.3.0),^[^ ^40^ ^]^ wtdbg2 (v2.5),^[^ ^41^ ^]^ and Canu (v2.1.1).^[^ ^42^ ^]^ Redundant sequences from the Canu assembly were removed using Purge_Dups (v1.2.5).^[^ ^43^ ^]^ The contigs generated by Canu were then scaffolded based on continuity and completeness (Figure S3 and Table S2, Supporting Information) using the 3D‐DNA pipeline,^[^ ^44^ ^]^ with Hi‐C data preprocessed by fastp (v0.20.1).^[^ ^45^ ^]^ The genome assembly was manually curated using PacBio long reads, telomere locations, Hi‐C signals, and synteny information with the EquCab3.0 reference genome, aided by Juicebox Assembly Tools (v1.11.08).^[^ ^46^ ^]^ The curated genome, along with the mitochondrial genome generated by GetOrganelle (v1.7.5),^[^ ^47^ ^]^ was polished through two rounds of PacBio long reads using the Arrow algorithm and two rounds of Illumina short reads using Pilon (v1.24).^[^ ^48^ ^]^ Chromosome numbers were assigned based on the alignment with EquCab3.0 (Figure S5, Supporting Information). Throughout the genome profiling stage, the assembly quality was evaluated using BUSCO (v5.2.2, mammalia_odb10),^[^ ^49^ ^]^ Merqury (v1.3),^[^ ^50^ ^]^ MUMmer (v4.0.0rc1),^[^ ^51^ ^]^ and QUAST (v5.0.2).^[^ ^52^ ^]^

### Genome Annotation

Repeat sequences were identified using a combination of homology‐based and de novo approaches. The homology‐based method employed the RMBlast search engine (v2.9.0, http://www.repeatmasker.org/rmblast/) with the transposable element (TE) databases from Dfam (v3.2, https://dfam.org) and Repbase (v20181026).^[^ ^53^ ^]^ De novo prediction was performed using RepeatModeler (v2.0.1),^[^ ^54^ ^]^ which integrated tools such as TRF (v4.09),^[^ ^55^ ^]^ RECON (v1.08),^[^ ^56^ ^]^ RepeatScout (v1.0.6),^[^ ^57^ ^]^ and LTR_Retriever (v2.9.0).^[^ ^58^ ^]^ The merged TE library was used for repeat sequence identification with RepeatMasker (v4.1.1, http://www.repeatmasker.org).

For genome annotation, an optimized iterative approach using MAKER3 (v3.01.03) was employed (Figure S8, Supporting Information).^[^ ^59^, ^60^ ^]^ The repeat library generated above was used to mask the genome. Evidence for gene annotation included protein sequences from six species (*Bos taurus*, *E. caballus*, *Homo sapiens*, *Mus musculus*, *Ovis aries*, and *Sus scrofa*), retrieved from UniProt (https://www.uniprot.org/) and transcripts from 14 tissues (adipose, cerebellum, cerebrum, dorsal muscle, heart, kidney, lung, large intestine, leg muscle, liver, stomach, small intestine, spleen, and testis) collected from the assembled Debao pony. Transcripts were obtained through RNA‐Seq using HISAT2 (v2.2.1)^[^ ^61^ ^]^ and StringTie (v2.1.4),^[^ ^62^ ^]^ as well as pooled Iso‐Seq using IsoSeq3 (v3.4.0, https://github.com/PacificBiosciences/IsoSeq) and minimap2 (v2.20).^[^ ^63^ ^]^ RNA‐Seq and Iso‐Seq data were sequenced on the Illumina NovaSeq 6000 platform and PacBio Sequel platform, respectively. The initial round of annotation utilized GeneMark‐ES (v4.64)^[^ ^64^ ^]^ for gene model training and prediction with evidence support. The second round involved training SNAP (v2006‐07‐28)^[^ ^65^ ^]^ and AUGUSTUS (v3.4.0)^[^ ^66^ ^]^ with predicted gene models, and the results were integrated to predict gene models. An additional iterative ab initio gene prediction step was implemented. Gene models without evidence support were rescued if they were annotated by BUSCO or InterProScan (v5.54‐87.0).^[^ ^67^ ^]^ Gene models that were absent compared to the NCBI Annotation Release 103 of EquCab3.0 were merged with the authors’ annotation dataset using GFF3toolkit (v2.1.0, https://github.com/NAL‐i5K/GFF3toolkit). The final MAKER3 gene models were manually curated using spliced alignment data from RNA‐Seq and Iso‐Seq with Apollo (https://cpt.tamu.edu/galaxy‐pub/).^[^ ^68^ ^]^ The accuracy of the genome annotation was assessed using annotation edit distance (AED). Functional annotation was assigned based on the best hit from DIAMOND (v2.0.14)^[^ ^69^ ^]^ alignment to the TrEMBL database. Motifs and domains of protein‐coding genes were determined using InterProScan. Gene ontology (GO) terms and KEGG pathways were assigned using the best‐match classification. Noncoding RNAs were predicted using RNAmmer (v1.2),^[^ ^70^ ^]^ tRNAscan‐SE (v2.0.7),^[^ ^71^ ^]^ and Infernal (v1.1.4).^[^ ^72^ ^]^

### Comparative Genomics Analysis

Protein‐coding genes from nine genomes, including horse (*E. caballus*: Thoroughbred and Debao pony), ass (*Equus asinus*), plains zebra (*Equus quagga*), black rhinoceros (*Diceros bicornis*), goat (*Capra hircus*), sheep (*O. aries*), cattle (*B. taurus*), pig (*S. scrofa*), and house mouse (*M. musculus*), were used. The longest protein sequences from alternative transcripts were selected to represent unique genes. Orthologous gene families were inferred using OrthoFinder (v2.5.4).^[^ ^73^ ^]^ Single‐copy gene families with sequences longer than 100 amino acids were aligned using MUSCLE (v5.1).^[^ ^74^ ^]^ The resulting protein sequence alignments were used to construct a maximum likelihood (ML) phylogenetic tree with RAxML (v8.2.12).^[^ ^75^ ^]^ Intermediate results from RAxML are shown in Table S19, Supporting Information. Divergence times were estimated using the MCMCTree program of PAML (v4.9),^[^ ^76^ ^]^ with prior divergence times obtained from the TimeTree database (http://www.timetree.org/), as detailed in Table S20, Supporting Information. Gene family expansions and contractions were detected using CAFE (v5.0).^[^ ^77^ ^]^ GO enrichment analysis was performed using the R package clusterProfiler (v4.6.0).^[^ ^78^ ^]^ To verify the contraction of olfactory receptor genes, olfactory receptor proteins from *E. asinus*, *E. quagga*, and *D. bicornis* longer than 300 amino acids were used as queries to search against the EquCab3.0 and DeBao1.0 genomes using TBLASTN (v2.15.0),^[^ ^79^ ^]^ with an e‐value cutoff of 1e‐10. Proteins extracted from each species’ genome were annotated using eggNOG‐mapper (v2.1.8).^[^ ^80^ ^]^ Matched sequences with 500 bp upstream and downstream were retrieved and used for TBLASTN searches. Best matches with percent identity ≥ 80% were defined as putative olfactory receptor genes. For further verification, paired‐end reads from the EquCab3.0 and DeBao1.0 assemblies were filtered with fastp. Qualified reads were aligned to the reference genomes of *E. asinus* and *E. quagga* using BWA‐MEM (v0.7.17‐r1188)^[^ ^81^ ^]^ with default parameters. Alignment files were sorted, and the depth for the olfactory receptor genes was calculated using SAMtools (v1.12).^[^ ^82^ ^]^ Putative lost olfactory receptor genes were identified within the constrained olfactory receptor gene family in horses.

### Resequencing and Variant Calling

Blood samples were collected from ten Debao ponies, ten Baise horses, and six Warmbloods. Genomic DNA was extracted using the DNeasy Blood & Tissue Kit (Qiagen, Beijing, China), and DNA integrity was assessed using gel electrophoresis. Paired‐end sequencing libraries were prepared and sequenced on an Illumina NovaSeq 6000 platform, generating 150‐bp reads with a target depth of 10× coverage. Publicly available whole‐genome sequencing (WGS) data for 455 domestic horses, 15 Przewalski's horses, 5 domestic donkeys, and 1 Somali wild ass (*Equus africanus somaliensis*) were downloaded from NCBI. Detailed information for all 502 individuals is provided in Table S7, Supporting Information.

Raw WGS data underwent quality control using fastp. Clean reads were mapped to the assembled Debao pony genome using BWA‐MEM with default parameters (Table S4, Supporting Information). The mapped reads were sorted and converted to BAM format using SAMtools. Variations were detected using the GATK (v4.2.0.0) germline short variant discovery pipeline (https://gatk.broadinstitute.org/). Duplicate reads were identified using the MarkDuplicates module. Variants were called via local re‐assembly of haplotypes using the HaplotypeCaller module. Raw genomic variant call format (GVCF) files were merged using the GenomicsDBImport module, and joint genotyping was performed using the GenotypeGVCFs module. The resulting variant calls (SNPs and indels) were subjected to hard filtering based on specific parameter thresholds: QD < 2.0 || MQ < 50.0 || FS > 60.0 || SOR > 3.0 || MQRankSum < −5.0 || ReadPosRankSum < −5.0 || QUAL < 30.0 (Figure S11, Supporting Information). The SNP dataset was annotated using SnpEff (v5.1d).^[^ ^83^ ^]^

### Population Genetics Analysis

Population genetic analysis was conducted using the dataset of horse samples (Table S7, Supporting Information). To ensure the reliability of breed records and lineage purity, potential crossbred samples were excluded based on inconsistencies in population structure. SNP filtering was performed using PLINK (v1.90b6.21)^[^ ^84^ ^]^ with the following criteria: missing genotype rate per sample >0.08%, missing genotype rate per variant >2%, minor allele frequency (MAF) <0.01, Hardy–Weinberg equilibrium *p*‐value < 0.000001, and SNPs pruned in linkage disequilibrium (LD) blocks (window size: 50; step size: 5; *r* ^2^ threshold: 0.5). Principal component analysis (PCA) was performed using GCTA (v1.93.2beta)^[^ ^85^ ^]^ with default settings, excluding Przewalski's horses. For phylogenetic analysis, the *P*‐distance matrix was calculated using VCF2Dis (https://github.com/BGI‐shenzhen/VCF2Dis). Based on the *P*‐distance matrix, a neighbor‐joining (NJ) phylogenetic tree was constructed using the fneighbor command of EMBOSS (v6.6.0.0).^[^ ^86^ ^]^ The tree was rooted on Przewalski's horses and visualized using the R package ggtree (v3.6.2).^[^ ^87^ ^]^ Ancestry and population structure were analyzed using ADMIXTURE (v1.3.0),^[^ ^88^ ^]^ with *K* values ranging from 4 to 9. In the SNP pruning step, the parameters used were window size: 50, step size: 10, and *r* ^2^ threshold: 0.1.

### Y Chromosome Haplotype

Y chromosome analysis was performed on male individuals (Table S7, Supporting Information). The sex of each individual was determined based on the read coverage of the Y chromosome, resulting in the identification of 201 male horses with Y chromosome coverage exceeding 94% (Table S11, Supporting Information). The sex determination results were further validated using the Rx and Ry metrics for accuracy.^[^ ^89^, ^90^ ^]^ SNPs specific to the Y chromosome were extracted, and heterozygous sites were excluded to minimize false positives within the male‐specific region of the Y chromosome (MSY). The remaining SNPs were filtered using specific criteria: missing genotype rate per sample >0.08%, missing genotype rate per variant >2%, and MAF <0.005. A phylogenetic tree rooted on Przewalski's horses was constructed based on the *P*‐distance matrix generated with VCF2Dis and the fneighbor command of EMBOSS. For haplotype analysis, variant sites with missing genotypes were excluded and haplotypes were identified using DnaSP (v6.12.03).^[^ ^91^ ^]^ Haplotype networks were subsequently constructed using PopArt (v1.7).^[^ ^92^ ^]^

### Genome Evolution Analysis

Genome evolution analysis included a dataset of horse and donkey samples (Table S7, Supporting Information).^[^ ^93^ ^]^ SNPs were filtered using PLINK with the following criteria: missing genotype rate per sample >0.8%, missing genotype rate per variant >2%, and MAF <0.001. A Norwegian Fjord Horse was included in maximum‐likelihood phylogenetic tree construction and outgroup *f3*‐ and *f4*‐statistics calculations (Table S21, Supporting Information).

Historical population relationships were inferred by constructing the ML phylogenetic tree and calculating outgroup *f3*‐statistics. The ML tree was built using TreeMix (v1.13),^[^ ^94^ ^]^ with variants present in all individuals. SNPs were further pruned with specific parameters: window size: 50, step size: 10, and *r* ^2^ threshold: 0.2. This resulted in a dataset of 1 52 90 640 autosomal SNPs, which were transformed into TreeMix input format using the plink2treemix.py script. The ML tree was rooted on *E. africanus somaliensis* with 1000 bootstrap replicates. Shared genetic drift between pairs of populations was quantified by calculating the *f3*‐statistics in the form *f3*(X, Y; outgroup), where X and Y represent all possible pairwise combinations of domestic horse breeds, and the outgroup is *E. africanus somaliensis*. The *f3*‐statistics were computed with 5 73 26 131 autosomal SNPs using the R package ADMIXTOOLS 2 (v2.0.0).^[^ ^95^ ^]^

LD decay patterns were measured separately for each breed (with ≥ 8 individuals) in two groups (Tables S7 and S8, Supporting Information): tall horses (Oldenburger, Holsteiner, Hanoverian horse, and Warmblood) and small ponies (Baise horse, Shetland pony, Debao pony, and Falabella). The LD decay analysis was performed using PopLDdecay (v3.42).^[^ ^96^ ^]^

A 2D PHATE embedding was generated using the top 133 principal components from the PCA as input in the R package phateR (v1.0.7),^[^ ^97^ ^]^ which exhibited a clear hierarchical structure and transition state trajectories. The parameters used were: ndim = 2, knn = 5, decay = 40, gamma = 1, t = auto, and seed = 1234.

Gene flows among pony populations were inferred with TreeMix using representative populations (Przewalski's horse, Debao pony, Ningqiang pony, Yakutian horse, Shetland pony, Icelandic horse, Mongolian horse, Friesian horse, Connemara pony, Holsteiner, and Arabian horse). The SNPs were filtered as described in the previous TreeMix procedure. The topology was rooted on *E. africanus somaliensis* with 1000 bootstrap replicates. The TreeMix model was run with 0–30 migration events and 10 replicates. Optimal migration edges were inferred using the R package optM (v0.1.6) with 1000 bootstraps performed using the R package BITE (v1.2.0008).^[^ ^98^ ^]^

The *f4*‐statistics were calculated to assess the genetic relationships between populations using autosomal SNPs from the *f3*‐statistics analysis. The *f4*‐statistics were modeled as *f4*(P1, P2; P3, outgroup) using the R package ADMIXTOOLS 2, where P1, P2, and P3 represent all possible permutations of domestic horse breeds, and the outgroup is *E. africanus somaliensis*. A relationship network was constructed by integrating all possible combinations of *f4*‐statistics. Distances between edges were calculated using the following formulas:

(1) $$disf4\left(a,b\right)=\sum_{i=1}^{n}f4\left(breed_{a},breed_{i};breed_{b},outgroup\right)/n$$

(2) $$\mathrm{dis}\mathrm{edge}\left(a,b\right)=\left[\mathrm{disf}4\left(a,b\right)+\mathrm{disf}4\left(b,a\right)\right]/2$$

where *disf4*(*a*, *b*) represents the directional distance from breed*_a_* to breed*_b_*, *f4*(*a*, *i*; *b*, *outgroup*) represents the specific *f4*‐statistics formulation, *n* represents the number of horse breeds included in the analysis, and *disedge*(*a*, *b*) represents the non‐directional distance between *breed*_a_ and *breed*_b_. Breeds geographically close to the Yakutian horse were not included in the pony‐related network.

### Identification of Height‐Related Genes

Identification of height‐related genes involved a comprehensive analysis of horse and pony samples (Tables S7 and S8, Supporting Information). The dataset included 42 horse individuals from tall horse breeds (Shire horse, Clydesdale horse, Percheron, Oldenburger, Holsteiner, Württemberger, Irish Sport Horse, Hanoverian horse, Dutch Warmblood, Belgian Warmblood, Westphalian horse, Bavarian Warmblood, Warmblood, and Trakehner) and 41 pony individuals from small pony breeds (Falabella, Miniature horse, Debao pony, Shetland pony, and Ningqiang pony). To exclude genetically related samples, individuals with genomic relationship matrix (GRM) values above 0.3, calculated by GCTA, were filtered out.

To detect selective signals, scans for selection signatures were performed using fixation index (*F*_ST_), nucleotide diversity (π) ratio (*θ*π_pony_/*θ*π_horse_), and cross‐population extended haplotype homozygosity (XP‐EHH) between ponies and horses. Genetic statistics of *F*_ST_ and *θ*π were calculated genome‐wide using 10 kb non‐overlapping sliding windows with VCFtools (v0.1.16).^[^ ^99^ ^]^ The variant dataset was phased using Beagle (v5.4).^[^ ^100^ ^]^ The XP‐EHH analysis was conducted on bi‐allelic SNPs using selscan (v1.2.0),^[^ ^101^ ^]^ with ponies designated as the query population and horses designated as the reference population (pony vs horse). Positive XP‐EHH scores indicate selection signals in ponies, whereas negative scores indicate selection in horses. Averaged XP‐EHH scores were calculated with 10 kb non‐overlapping sliding windows. For each metric, genomic regions falling within the top 1% of values were considered statistically significant. Regions identified as significant in at least two of these metrics were designated as selective sweeps. Candidate genes were selected based on their presence within these regions and their known or potential roles in regulating body size in horses.

Hi‐C data analysis was conducted using the HiCExplorer (v3.7.2) pipeline.^[^ ^102^ ^]^ To ensure robust results, Hi‐C data from both the Debao pony assembly in this study and the EquCab3.0 assembly were initially merged. Hi‐C reads preprocessed by fastp were mapped to the DeBao1.0 genome using BWA‐MEM with specific parameters (−A1, −B4, −E50, and −L0). Aligned reads in BAM format were filtered, and a contact matrix with a bin size of 10 kb was created using the hicBuildMatrix function. The Hi‐C matrix was then normalized and corrected using the hicNormalize and hicCorrectMatrix functions with a threshold of 1.5. Topologically associating domains (TADs) were identified using the hicFindTADs function.

Regulatory elements were annotated using ChIP‐seq data obtained from the Functional Annotation of Animal Genomes (FAANG) database (https://data.faang.org). Complete archived ChIP‐seq data for horses are shown in Table S22, Supporting Information. Specifically, histone marks including H3K4me1, H3K4me3, H3K27ac, and H3K27me3 were retrieved from the diaphysis of the metacarpal bone and processed using Trim Galore (v0.6.7, https://www.bioinformatics.babraham.ac.uk/projects/trim_galore/). Clean reads were subsequently mapped to the DeBao1.0 genome using Bowtie 2 (v2.4.5)^[^ ^103^ ^]^ with default parameters. Non‐aligned and poorly mapped reads (MAPQ < 10) were filtered out using SAMtools. The aligned reads were sorted, and duplicate alignments were marked using SAMtools. ChIP‐seq peak calling was performed using MACS2 (v2.2.7.1).^[^ ^104^ ^]^ Bigwig files were generated for track visualization with deepTools (v3.5.1).^[^ ^105^ ^]^

Runs of homozygosity (ROH) were analyzed using the –homozyg function in PLINK with specific parameters (Table S7, Supporting Information): ROH ratio (kb/variant) ≤50, maximum between‐variant distance within an ROH ≤100, ROH length ≥500 kb, minimum number of SNPs in a ROH ≥50, maximum number of heterozygous calls in a window ≤1, scanning window size = 50 SNPs, and scanning window hit rate ≥0.05 (Figure S19, Supporting Information).

PhyloP scores were computed based on conservation scoring by phyloP for multiple alignments of placental mammals to the human genome,^[^ ^106^ ^]^ available at http://hgdownload.cse.ucsc.edu/goldenPath/hg19/phyloP46way/placentalMammals/. Briefly, the alignment was achieved by aligning the DeBao1.0 genome to the EquCab2.0 genome using minimap2. A chain file was created from minimap2 using transanno (v0.3.0, https://github.com/informationsea/transanno). Genome coordinates were then converted using the chain file created above and the one obtained from UCSC (https://hgdownload.soe.ucsc.edu/goldenPath/equCab2/liftOver/equCab2ToHg19.over.chain.gz) using liftOver (v447).^[^ ^107^ ^]^ Blocks with a distance of less than 10 bp were merged using BEDTools (v2.30.0).^[^ ^108^ ^]^

Fisher's exact test was conducted using BEDTools to evaluate the number of overlapping selected regions between Eastern pony versus horse and Western pony versus horse. The horse dataset remained consistent with that described earlier. Pony populations were categorized by geographic location into Eastern and Western groups. SNPs identified by XP‐EHH that were less than 100 bp apart were merged. To assess statistical significance, random shuffling of the selected regions was performed using BEDTools for permutation analysis.

Candidate regions were visualized using the R package plotgardener (v1.4.2).^[^ ^109^ ^]^ For most visualizations, the R package ggplot2 (v3.4.2) was utilized unless otherwise specified.

## Conflict of Interest

The authors declare no conflict of interest.

## Author Contributions

X.L., Z.W., and M.Z. contributed equally to this work. X.L., Z.T., Y.L., and G.Y. conceptualized the study. Z.W., M.Z., B.W., X.Q., K.Z., and M.P.C. conducted the investigations. S.T., J.Y., H.W., P.Y., and S.C. provided the study materials. X.L., Q.B., and S.H. curated the data. X.L., Z.W., and X.Y. developed the methodology. X.L., L.L., and P.Z. performed the statistical and computational analyses. X.L. prepared the data visualizations. Z.T., Y.L., and G.Y. supervised the research. X.L. wrote the original draft of the manuscript. X.L., J.J., H.X., L.F., and G.Y. reviewed and edited the manuscript. Z.W., Y.L., and G.Y. acquired funding.

## Supporting information



Supporting Information

Supporting Table 1

Supporting Table 2

Supporting Table 3

Supporting Table 4

Supporting Table 5

Supporting Table 6

Supporting Table 7

Supporting Table 8

Supporting Table 9

Supporting Table 10

Supporting Table 11

Supporting Table 12

Supporting Table 13

Supporting Table 14

Supporting Table 15

Supporting Table 16

Supporting Table 17

Supporting Table 18

Supporting Table 19

Supporting Table 20

Supporting Table 21

Supporting Table 22

## Data Availability

The data that support the findings of this study are openly available in NCBI at https://www.ncbi.nlm.nih.gov/bioproject/PRJNA1005486.
