# Supplementary material for: Genomic Insights into Post‐Domestication Expansion and Selection of Body Size in Ponies
Source: Adv Sci (Weinh). 2025 Feb 26;12(16):2413023. doi: 10.1002/advs.202413023 (PMC12021115; doi:10.1002/advs.202413023)
Supplement: Supplementary file 1 — Supporting Information [file ADVS-12-2413023-s015.docx]

Supporting Information

Title: Genomic Insights into Post-domestication Expansion and Selection of Body Size in Ponies

Xingzheng Li, Zihao Wang, Min Zhu, Binhu Wang, Shaohua Teng, Jing Yan, Haoyu Wang, Pengxiang Yuan, Shuwei Cao, Xiaolu Qu, Zhen Wang, Kai Zhan, Md. Panir Choudhury, Xintong Yang, Qi Bao, Sang He, Lei Liu, Pengju Zhao, Jicai Jiang, Hai Xiang, Lingzhao Fang, Zhonglin Tang*, Yuying Liao*, Guoqiang Yi*

This PDF file includes:

Figure S1 to S22


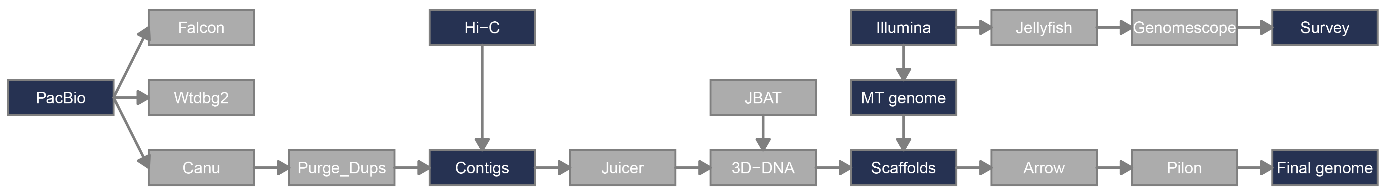


**Figure S1.** Workflow of the genome assembly pipeline. Flowchart chart of the genome assembly pipeline used to generate the DeBao1.0 assembly in this study. Dark rectangles represent raw input data or key outputs.


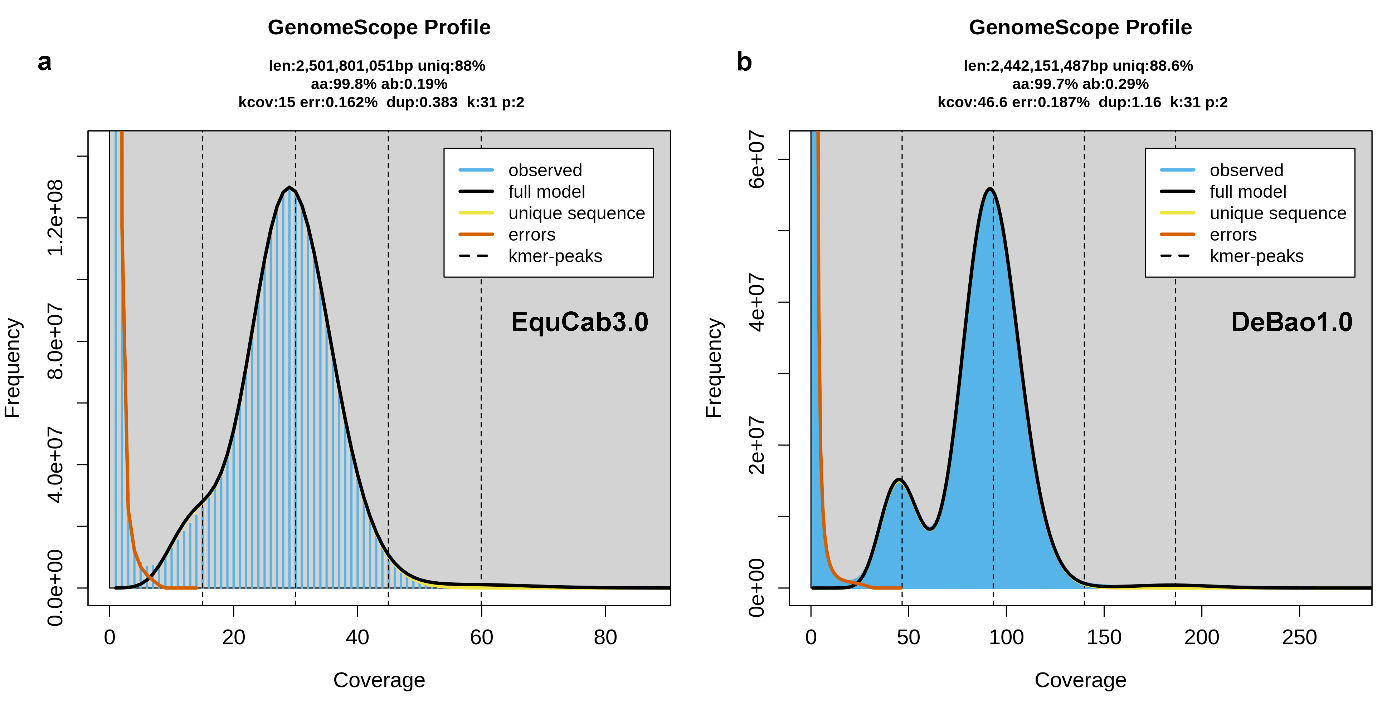


**Figure S2.** GenomeScope profiles generated from Illumina short-read data for *Equus caballus*. The figure presents the k-mer spectra and their corresponding fitted models for (a) the EquCab3.0 assembly and (b) the DeBao1.0 assembly.


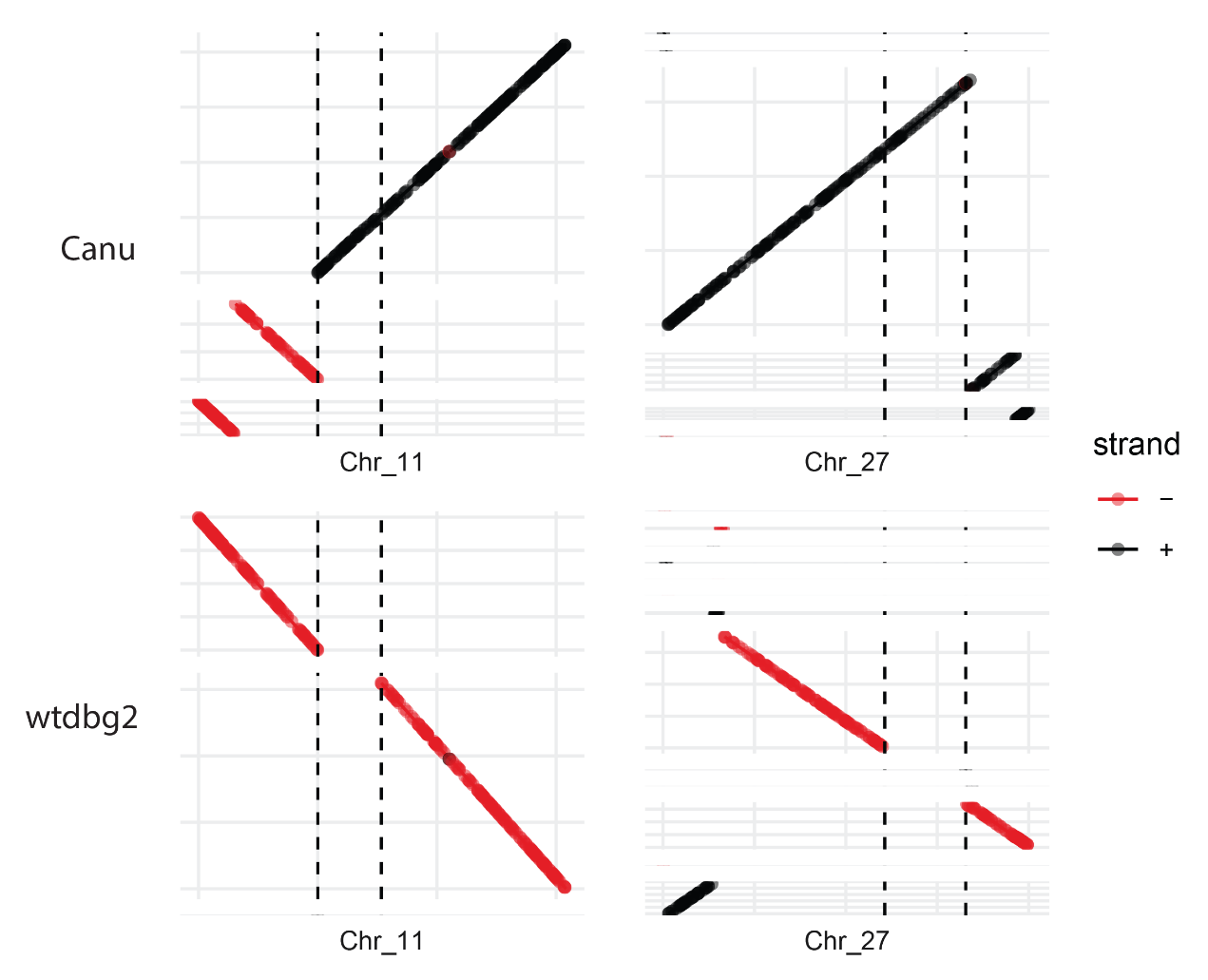


**Figure S3.** Comparison of primary assembly completeness achieved by Canu and wtdbg2. The primary assemblies were aligned to the EquCab3.0 genome using MUMmer. The x-axis represents EquCab3.0, and the y-axis represents primary contigs. Missing sequences in the wtdbg2 assembly are indicated between black dashed lines.


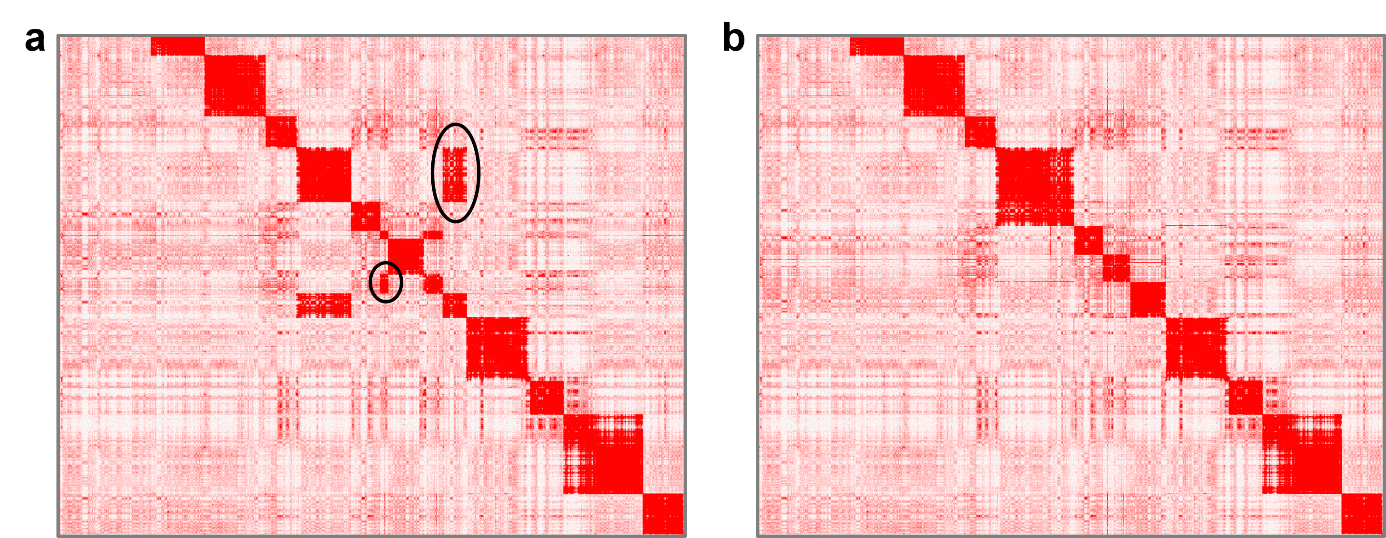


**Figure S4.** Correction of misassemblies using Hi-C data. (a) Hi-C map illustrating the pony assembly obtained by scaffolding without any editing, with misjoins indicated by black ellipses. (b) Hi-C map after the misjoins have been corrected.


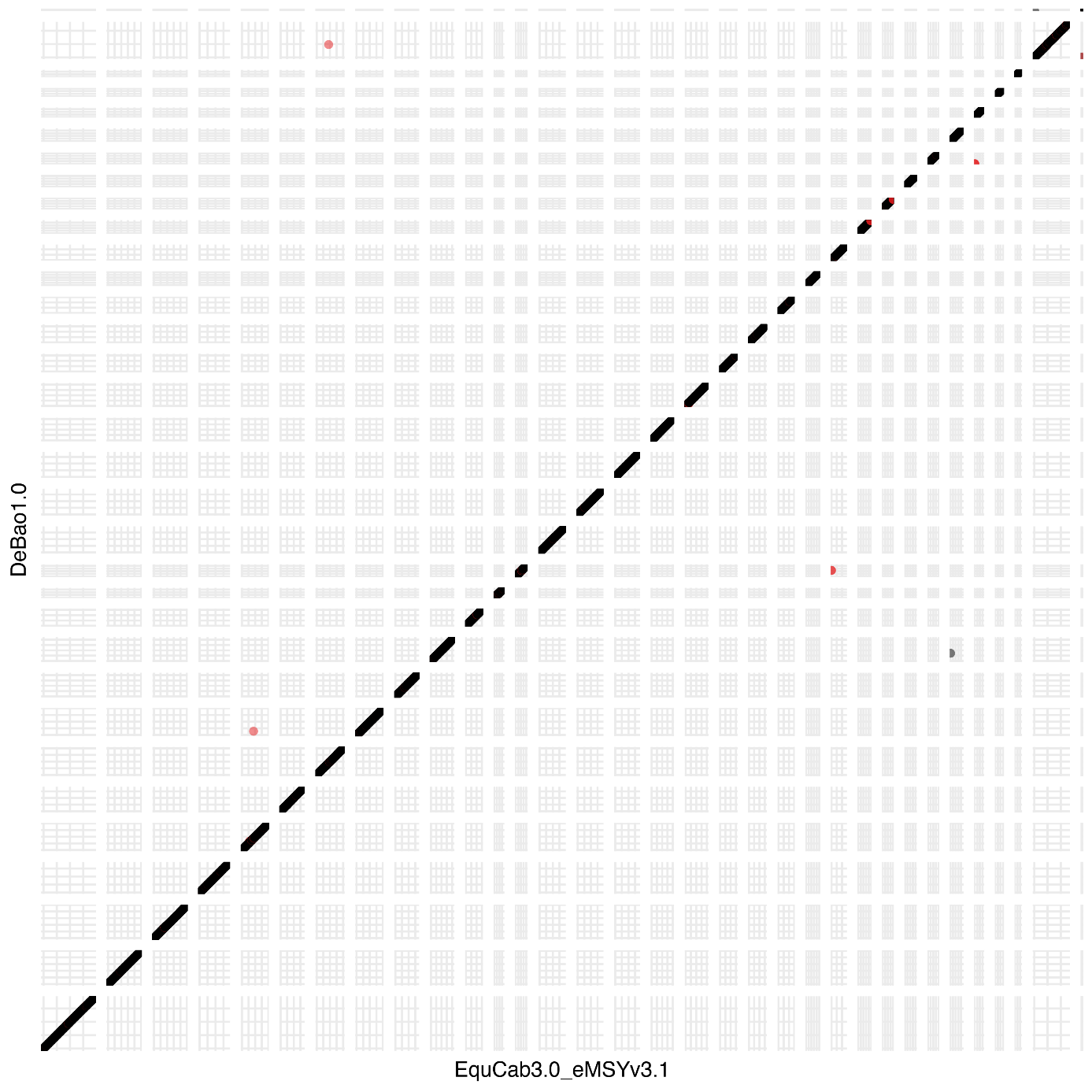


**Figure S5.** Synteny between DeBao1.0 and EquCab3.0 chromosomes. The Y chromosome assembly (eMSYv3.1) is appended to EquCab3.0. Black color represents alignment to the forward strand, and the red color represents alignment to the reverse strand.


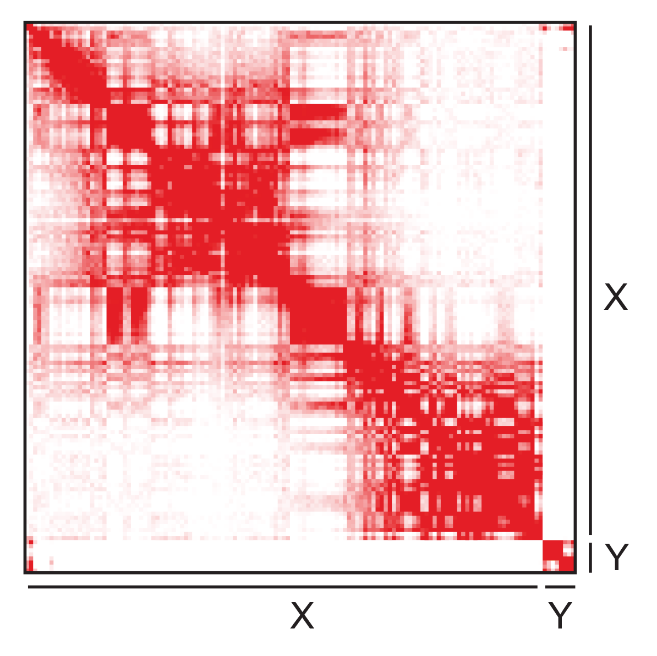


**Figure S6.** Hi-C chromatin interactions of the assembled sex chromosomes.


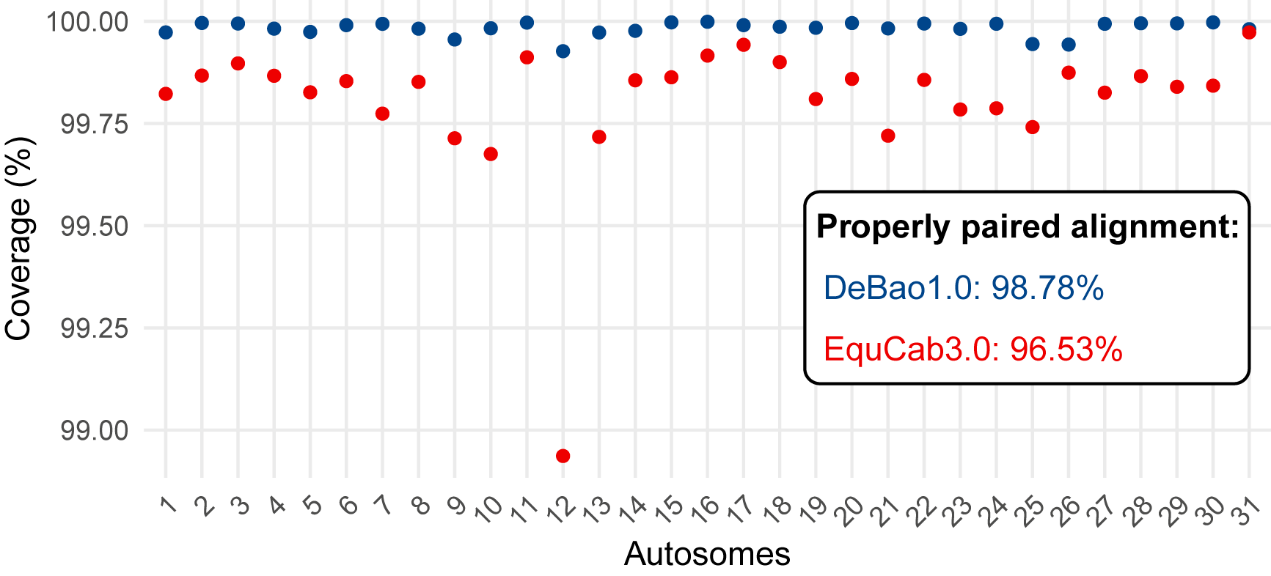


**Figure S7.** Alignment statistics of read coverage rate per autosome and the overall alignment rate for the DeBao1.0 and EquCab3.0 genome assemblies. Illumina paired-end (PE) reads used in generating each corresponding assembly were utilized for calculating these statistics.


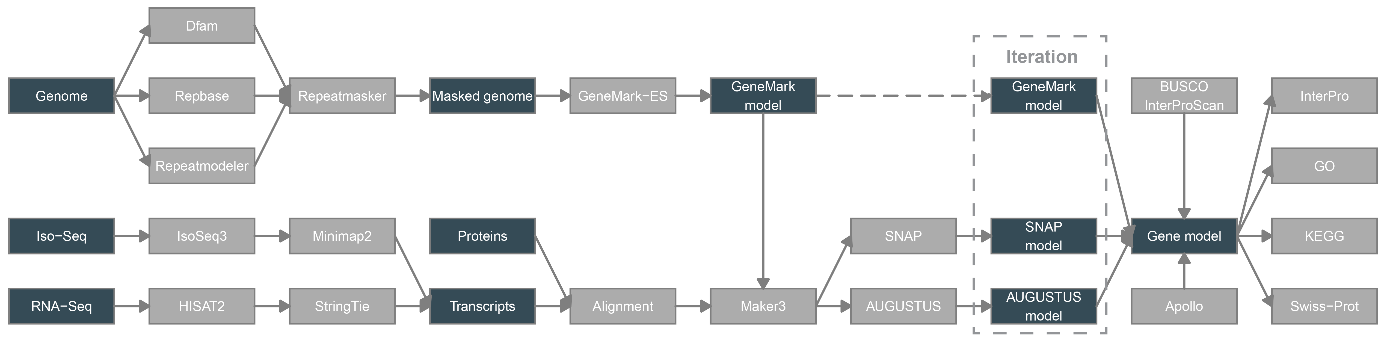


**Figure S8.** Workflow of the genome annotation pipeline. Flowchart chart of the genome annotation pipeline used to annotate the DeBao1.0 assembly in this study. Dark rectangles represent raw input data or key outputs.


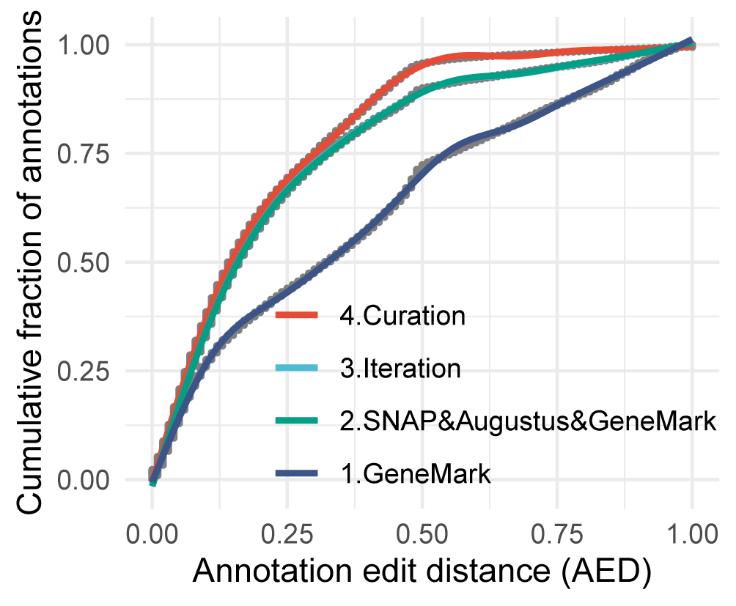


**Figure S9.** Annotation Edit Distance (AED) using iterative MAKER3 annotation to assess annotation accuracy.


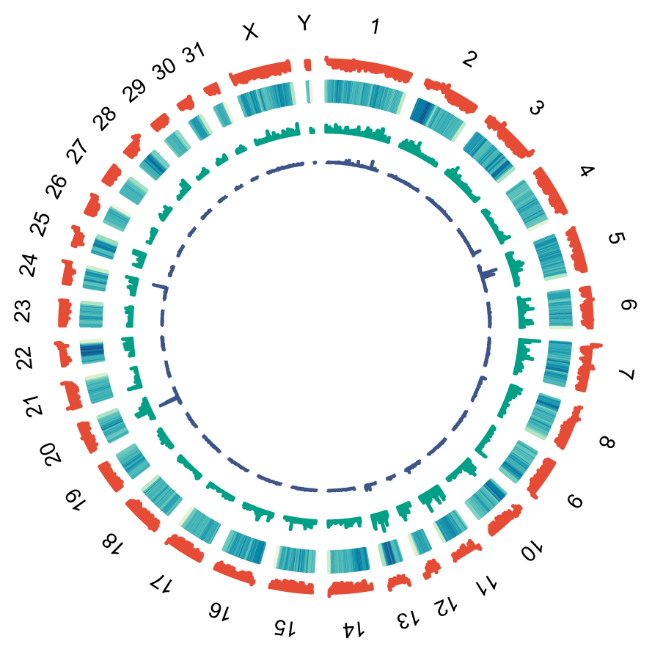


**Figure S10.** Landscape of the assembled pony genome. Visualization of chromosome names, GC content, repeat density, gene density, and noncoding RNA (ncRNA) density in different tracks from outer to inner.


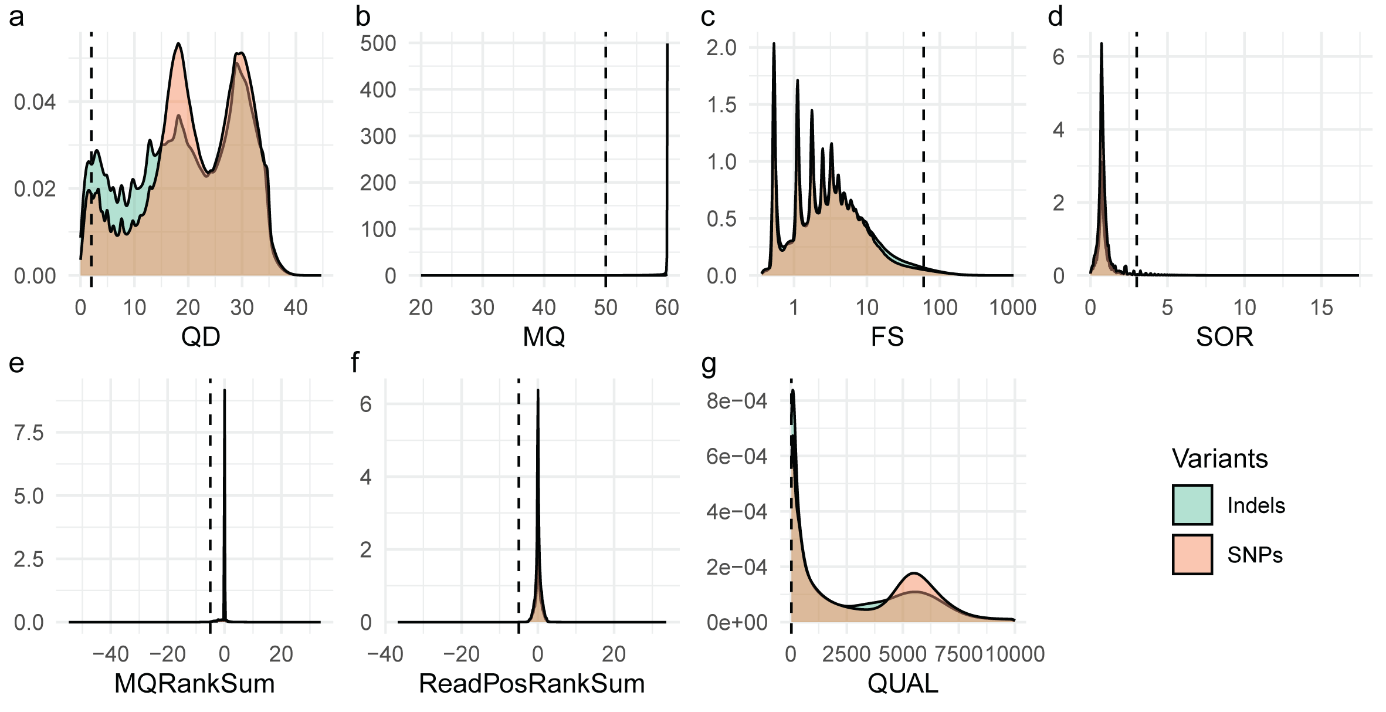


**Figure S11.** Distribution of hard-filtering parameters in GATK variant calling (SNPs & indels). Parameters include (a) QualByDepth (QD), (b) RMSMappingQuality (MQ), (c) FisherStrand (FS), (d) StrandOddsRatio (SOR), (e) MappingQualityRankSumTest (MQRankSum), (f) ReadPosRankSumTest (ReadPosRankSum), and (g) QUAL. The parameters used for filtering are indicated with black dashed lines.


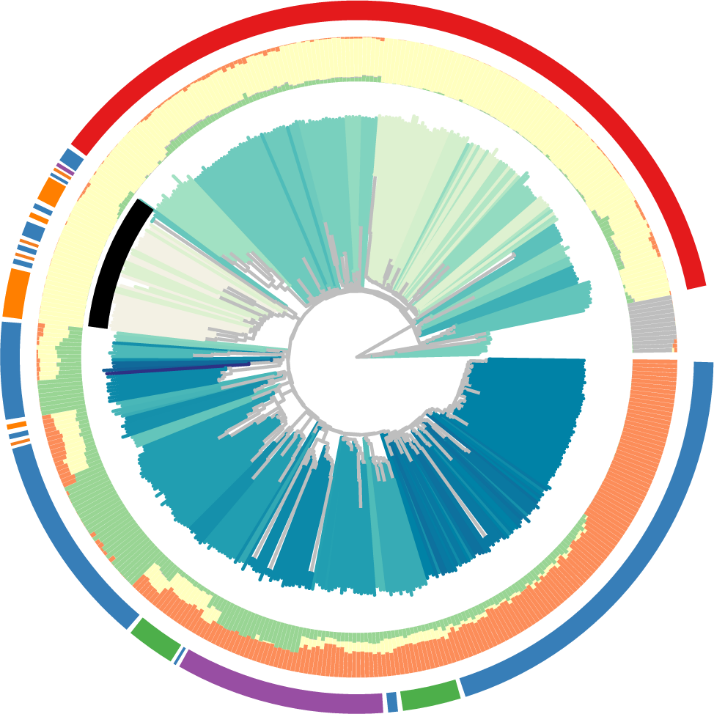


**Figure S12.** Neighbor-joining (NJ) tree (rooted on Przewalski's horses) alongside the admixture analysis (*K* = 4) for horse populations. The black inner section ring highlights the Western pony lineage, including the Shetland pony, Icelandic horse, Miniature horse, and Falabella. The middle circular ring represents the admixture structure. The outer section ring uses colors to indicate regions, while branch colors represent withers height. Colors in this figure are consistent with Figure 3.


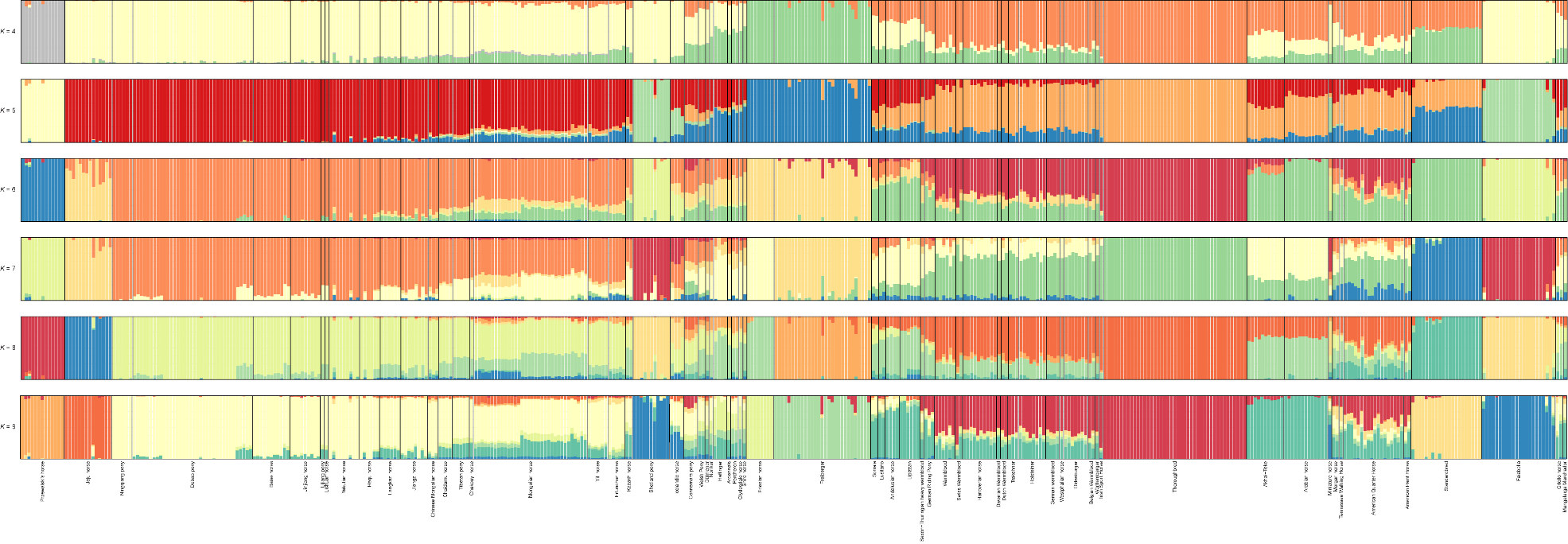


**Figure S13.** Admixture analysis of the horse population with *K* values ranging from 4 to 9.


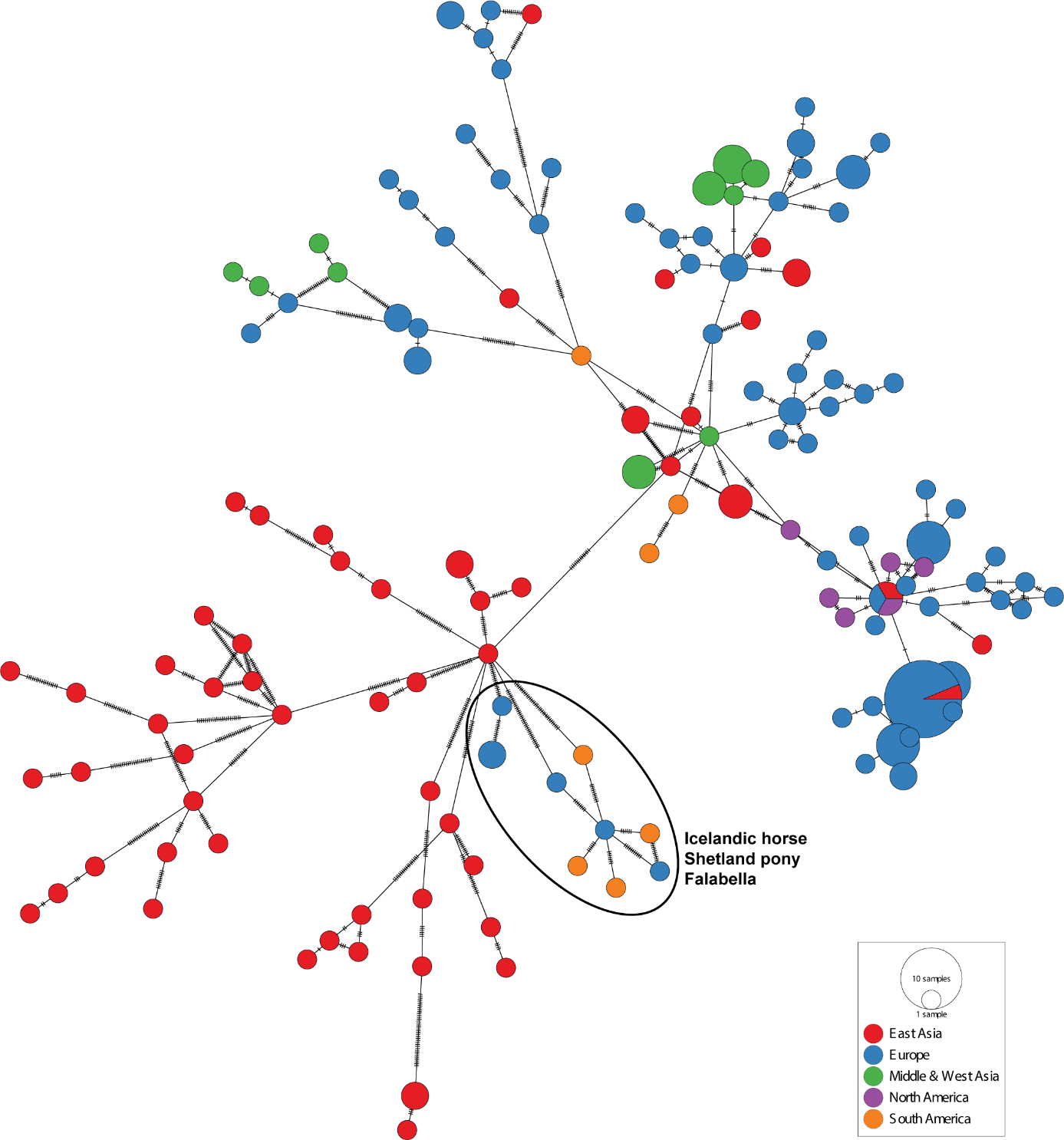


**Figure S14.** Haplotype network of Y chromosome variants among horse breeds (excluding Przewalski’s horse). The black ellipse highlights the Western pony lineage, encompassing the Icelandic horse, Shetland pony, and Falabella.


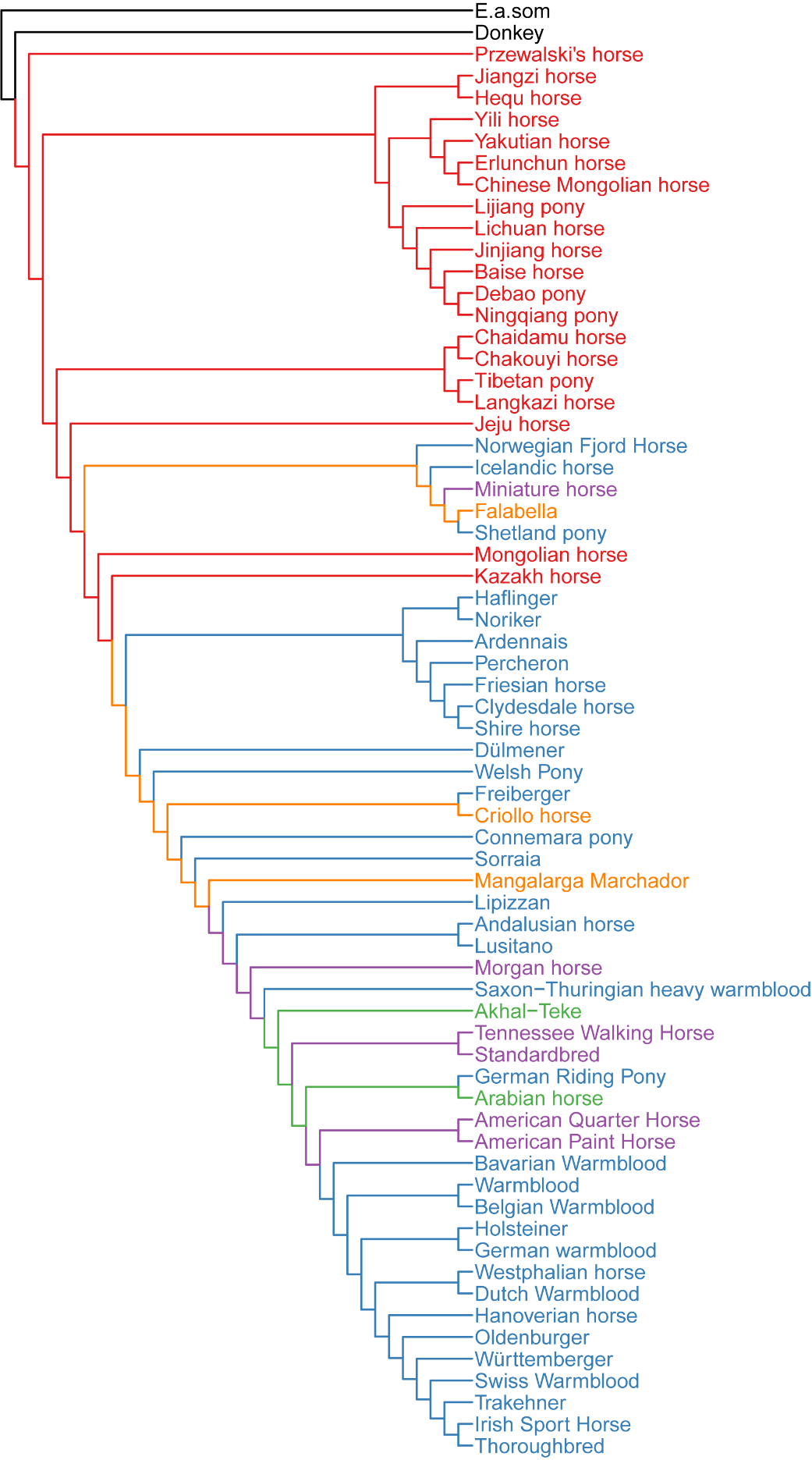


**Figure S15.** Phylogenetic relationship reconstruction using TreeMix. This figure corresponds to Figure 5A, illustrating the phylogenetic relationships among horse breeds.


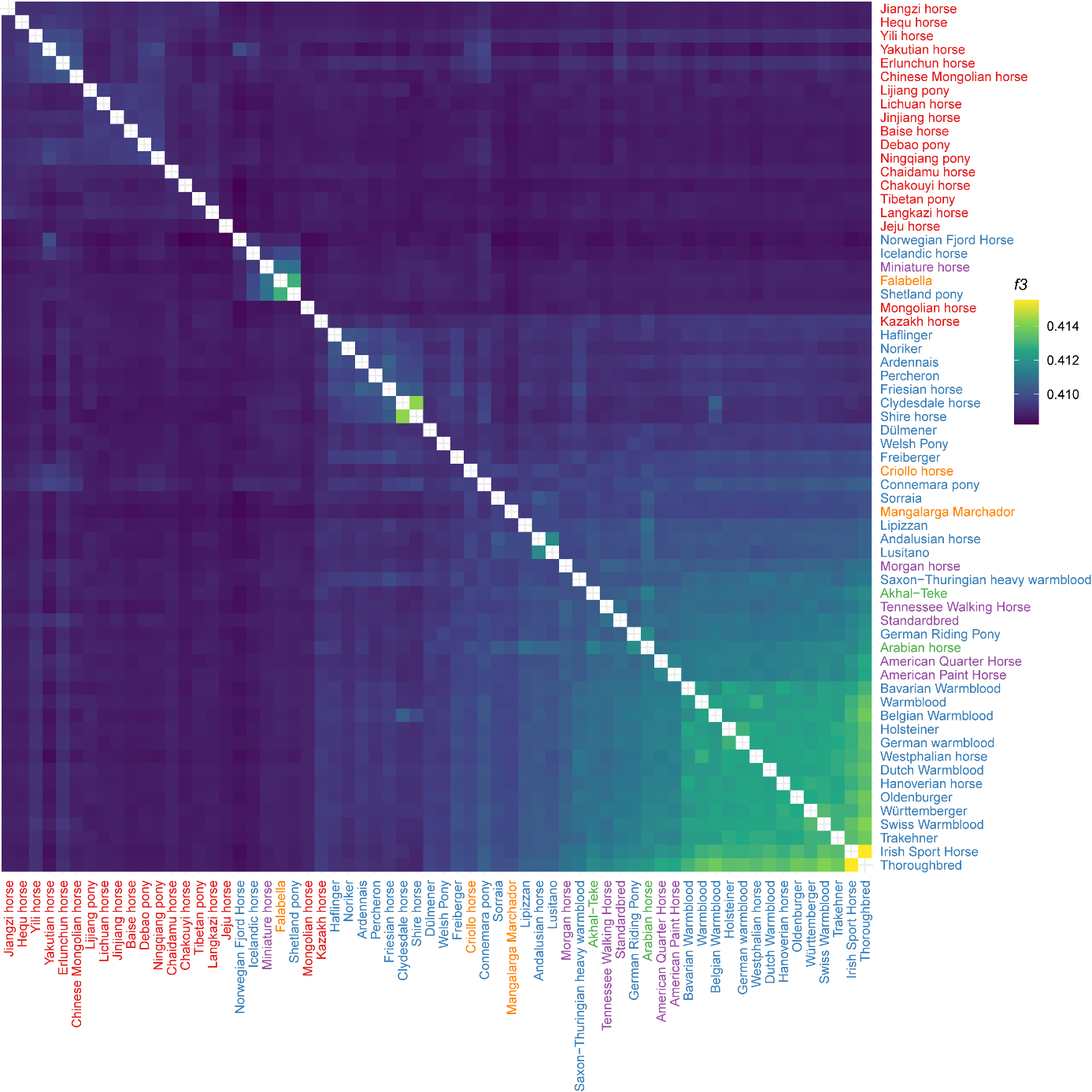


**Figure S16.** Heatmap displaying outgroup *f3*-statistics in the form of *f3*(*Equus caballus*, *Equus caballus*; *Equus africanus somaliensis*). This figure corresponds to Figure 5A.


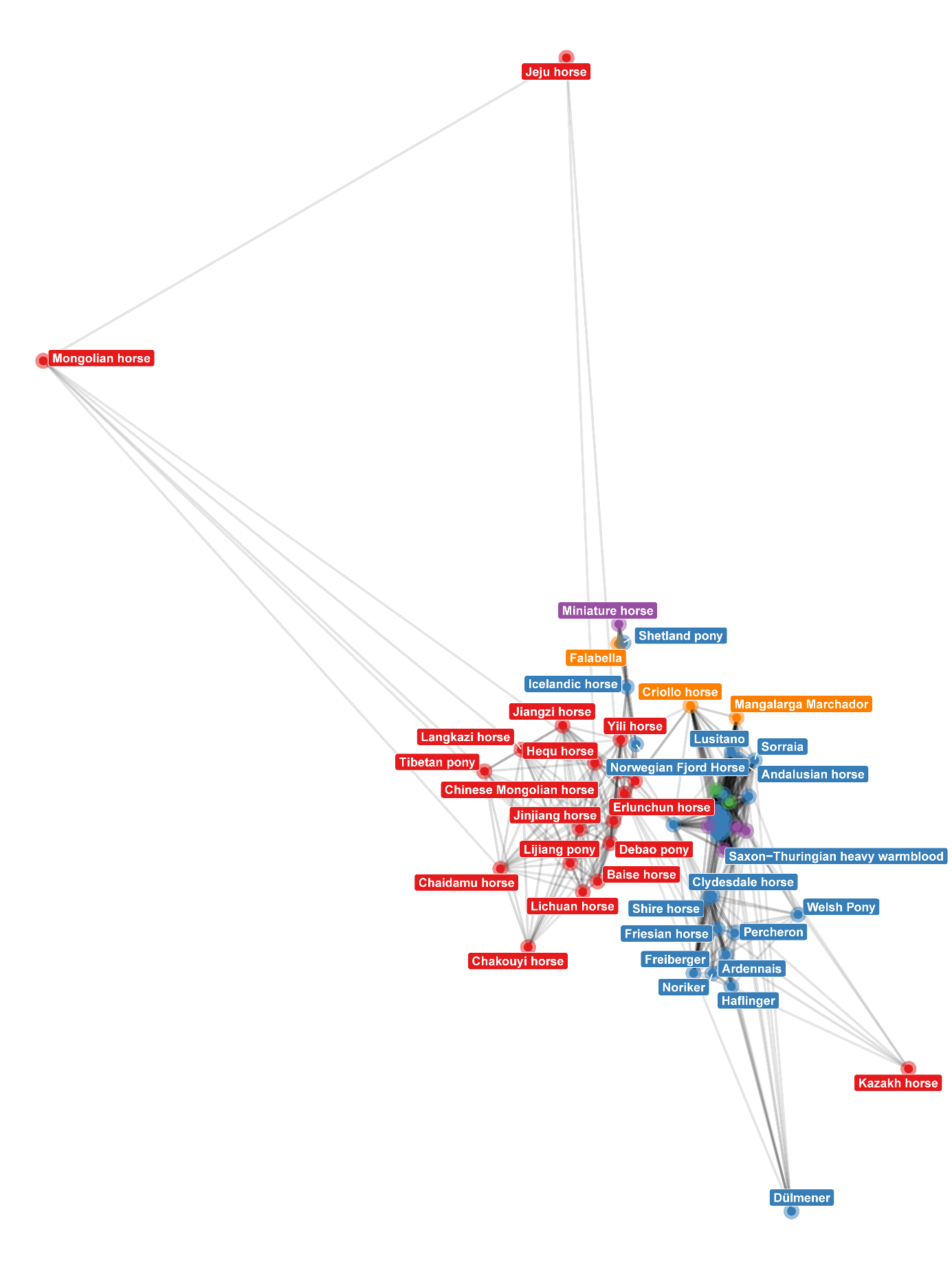


**Figure S17.** Relationship network based on all permutations of *f4*-statistics in the form of *f4*(*Equus caballus*, *Equus caballus*; *Equus caballus*, *Equus africanus somaliensis*). Line colors ranging from light to dark indicate genetic relationships from distant to close. The pony-related network shown in Figure 5E is derived from this network.


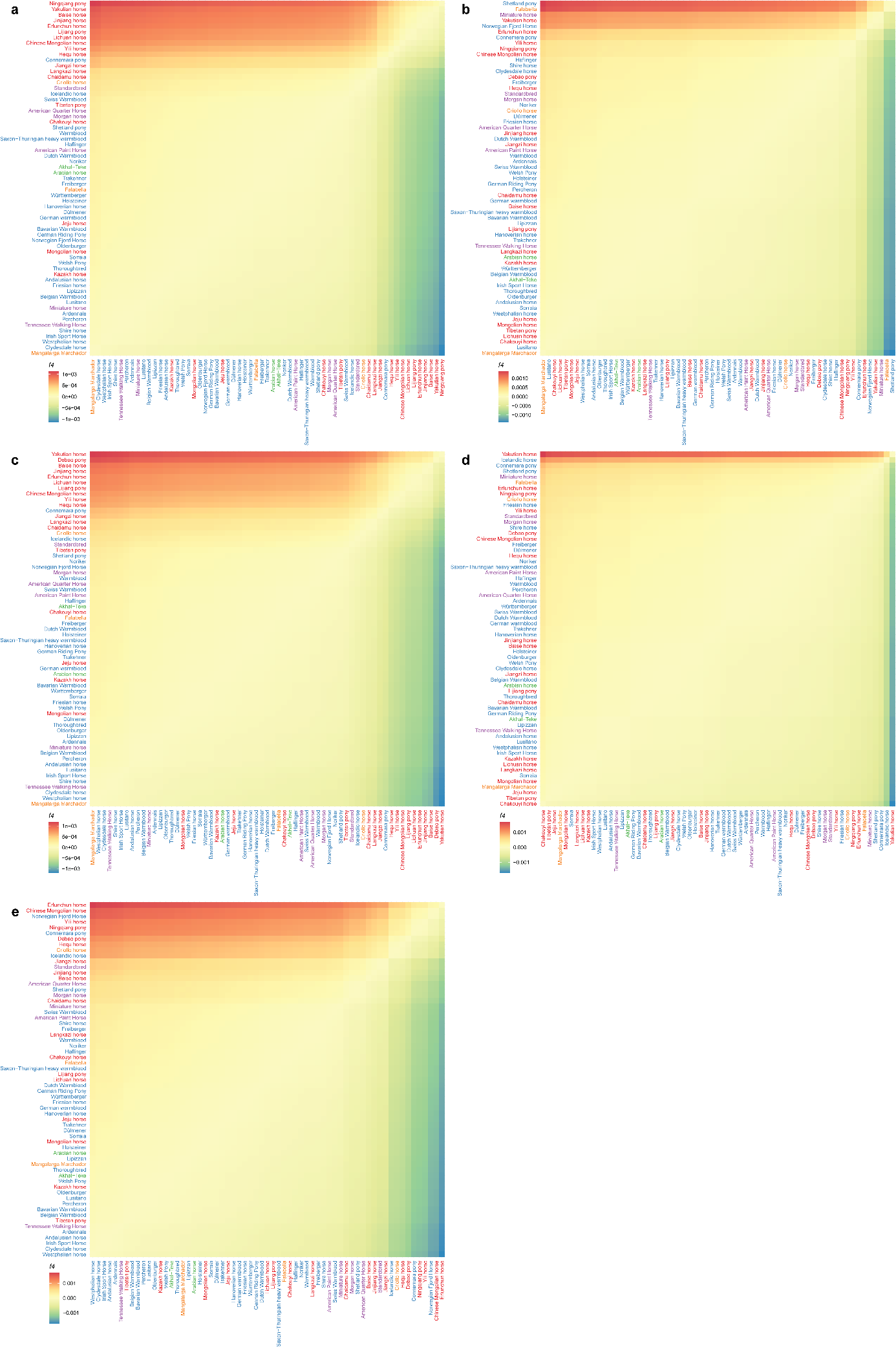


**Figure S18.** Heatmap displaying *f4*(P1*_Equus caballus_,* P2*_Equus caballus_,* P3*_Equus caballus_,* OG*_Equus africanus somaliensis_*) for key nodes in the pony relationship network. This figure corresponds to the nodes in Figure 5E, with (a) Debao pony, (b) Icelandic horse, (c) Ningqiang pony, (d) Norwegian Fjord Horse, and (e) Yakutian horse as P3. Each row represents P1, and each column represents P2.


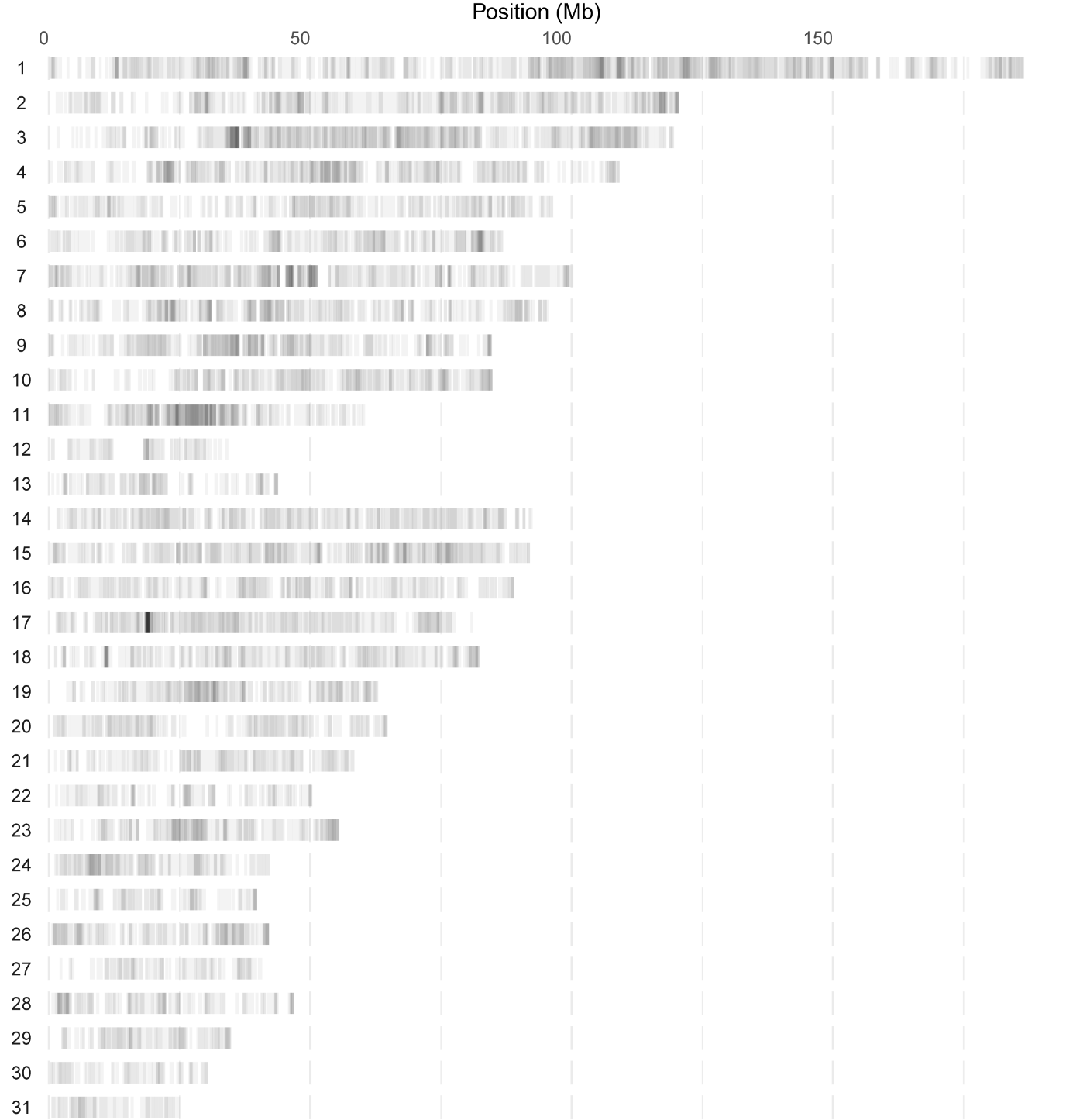


**Figure S19.** Genome-wide distribution of runs of homozygosity (ROH) across autosomes in pony-sized populations. The degree of darkness is proportional to the number of individuals with ROH.


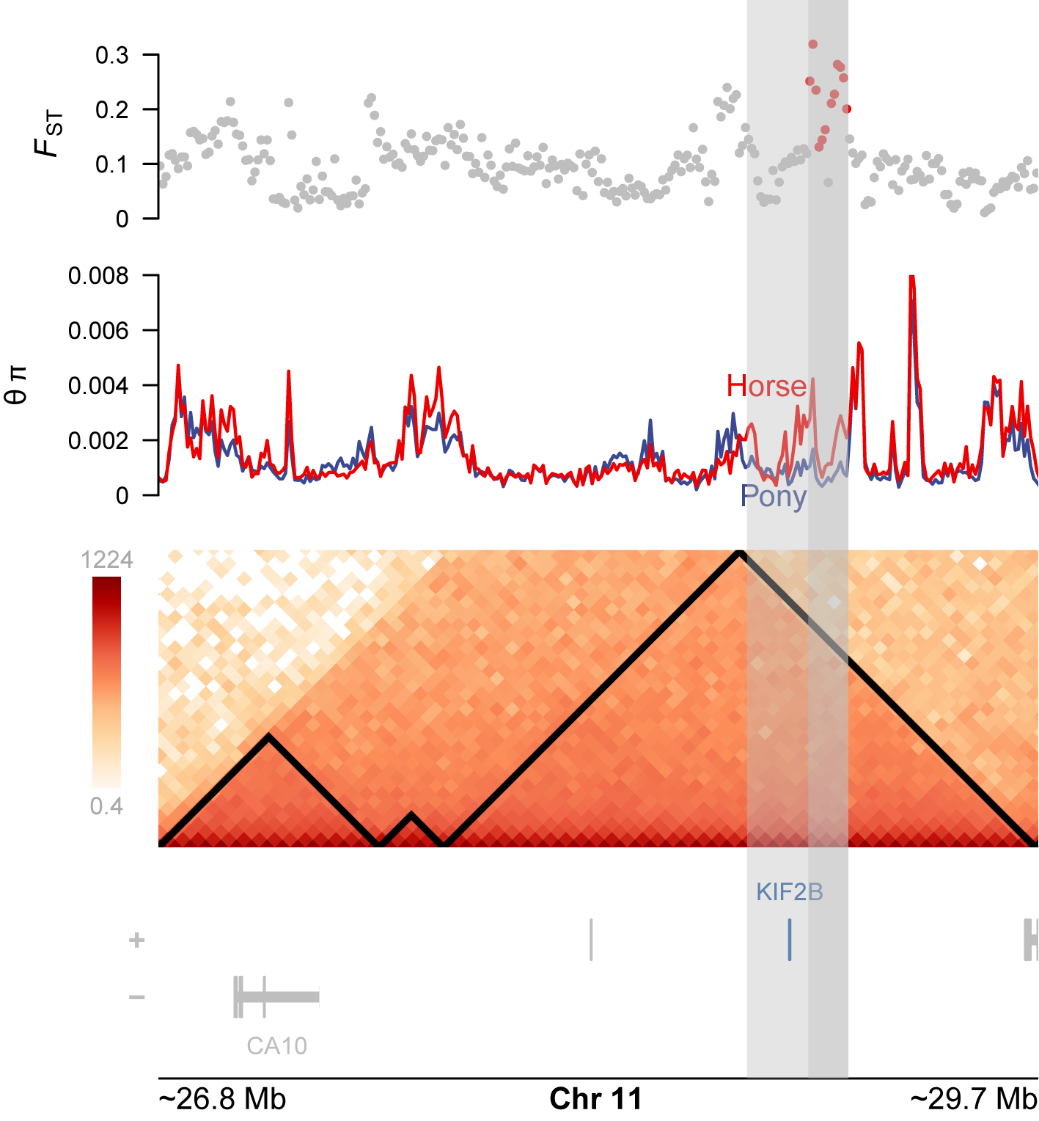


**Figure S20.** Candidate selective region on chromosome 11. From top to bottom: *F*_ST_, θπ, Hi-C matrix, and gene models. TADs are indicated with black triangles in the Hi-C matrix.


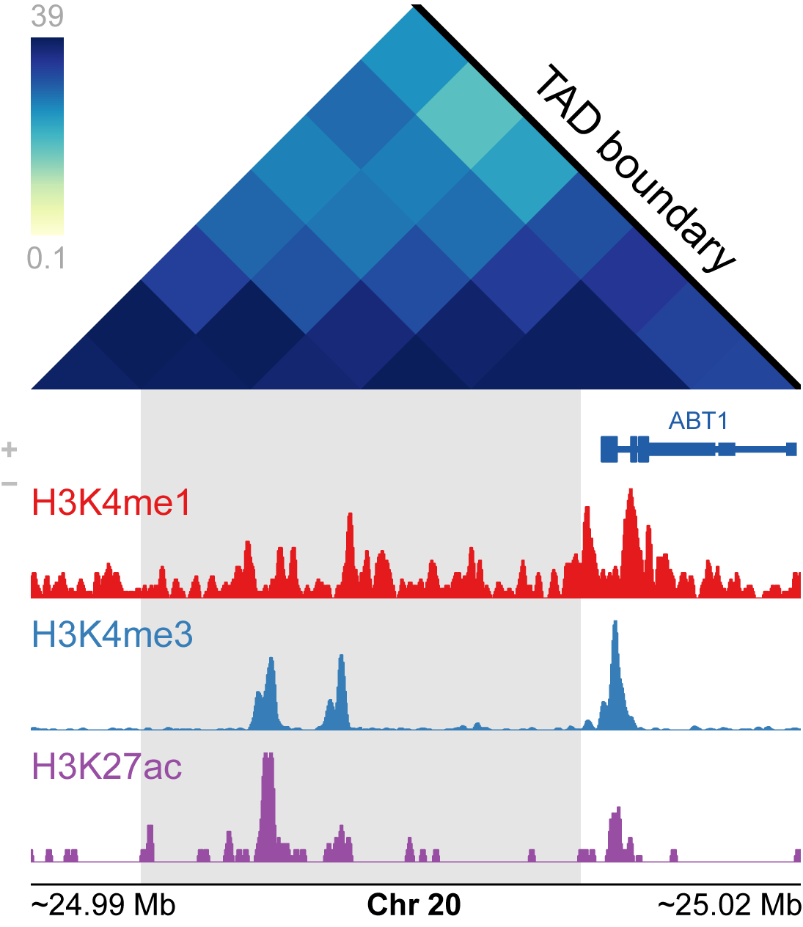


**Figure S21.** Candidate selective region on chromosome 20. From top to bottom: Hi-C matrix, gene models, and histone ChIP-seq signals (diaphysis of the metacarpal bone) signals. The TAD boundary is marked with a black line in the Hi-C matrix.


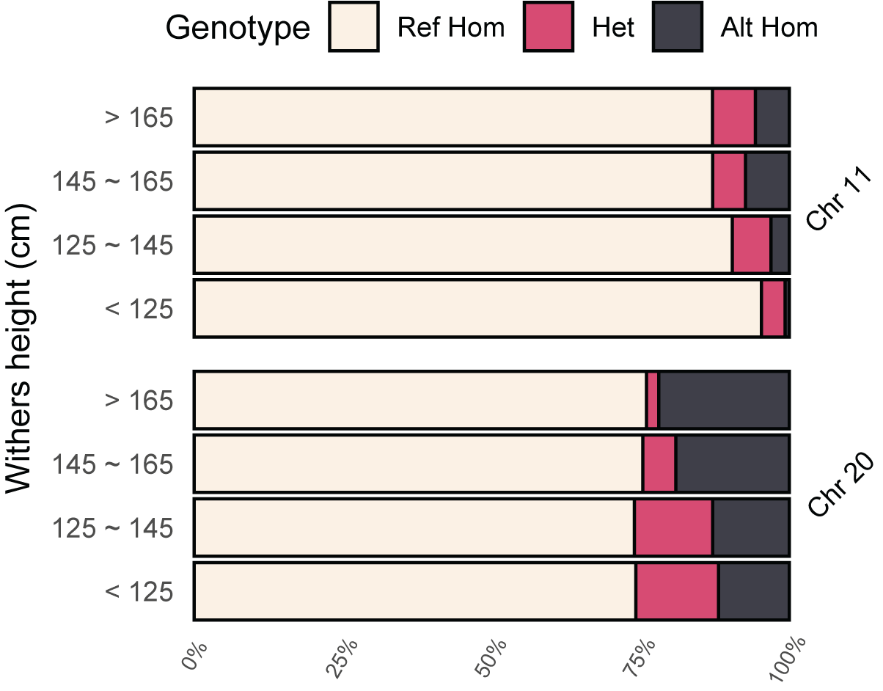


**Figure S22.** Genotype frequency of candidate regions at different withers heights on chromosomes 11 and 20. Bar plots showing the frequency of genotypes associated with body size across breeds of varying withers heights.
